# Generation of biallelic knock-out sheep via gene-editing and somatic cell nuclear transfer

**DOI:** 10.1038/srep33675

**Published:** 2016-09-22

**Authors:** Honghui Li, Gui Wang, Zhiqiang Hao, Guozhong Zhang, Yubo Qing, Shuanghui Liu, Lili Qing, Weirong Pan, Lei Chen, Guichun Liu, Ruoping Zhao, Baoyu Jia, Luyao Zeng, Jianxiong Guo, Lixiao Zhao, Heng Zhao, Chaoxiang Lv, Kaixiang Xu, Wenmin Cheng, Hushan Li, Hong-Ye Zhao, Wen Wang, Hong-Jiang Wei

**Affiliations:** 1 State Key Laboratory for Conservation and Utilization of Bio-Resources in Yunnan, Yunnan Agricultural University, Kunming 650201, China; 2 College of Animal Science and Technology, Yunnan Agricultural University, Kunming 650201, China; 3 College of Hetao, Bayannaoer 015000, China; 4 Inner Mongolia Zhong-Ke-Zheng-Biao Biotech Co., Ltd, Bayannaoer 015400, China; 5 State Key Laboratory of Genetic Resources and Evolution, Kunming Institute of Zoology, Chinese Academy of Sciences, Kunming 650223, China; 6 Reproductive & Developmental Laboratory, Southwest China Biodiversity Laboratory, Kunming 650203, China; 7 Key Laboratory Animal Nutrition and Feed of Yunnan Province, Yunnan Agricultural University, Kunming 650201, China; 8 Bayannaoer Livestock Improvement Station, Bayannaoer 015000, China

## Abstract

Transgenic sheep can be used to achieve genetic improvements in breeds and as an important large-animal model for biomedical research. In this study, we generated a TALEN plasmid specific for ovine *MSTN* and transfected it into fetal fibroblast cells of STH sheep. *MSTN* biallelic-KO somatic cells were selected as nuclear donor cells for SCNT. In total, cloned embryos were transferred into 37 recipient gilts, 28 (75.7%) becoming pregnant and 15 delivering, resulting in 23 lambs, 12 of which were alive. Mutations in the lambs were verified via sequencing and T7EI assay, and the gene mutation site was consistent with that in the donor cells. Off-target analysis was performed, and no off-target mutations were detected. *MSTN* KO affected the mRNA expression of *MSTN* relative genes. The growth curve for the resulting sheep suggested that *MSTN* KO caused a remarkable increase in body weight compared with those of wild-type sheep. Histological analyses revealed that *MSTN* KO resulted in muscle fiber hypertrophy. These findings demonstrate the successful generation of *MSTN* biallelic-KO STH sheep via gene editing in somatic cells using TALEN technology and SCNT. These *MSTN* mutant sheep developed and grew normally, and exhibited increased body weight and muscle growth.

Myostatin (MSTN) is a member of the transforming growth factor-β superfamily and plays a negative regulatory role in muscle differentiation and growth^1^,^2^. Previous studies have shown that the inhibition of *MSTN* expression results in a significant increase in muscle volume and mass, producing more meat in animals, which are known as double-muscle animals^1^,^3^,^4^. Genetic manipulations of the *MSTN* gene or the use of natural *MSTN* mutations for livestock meat production have great potential for increasing feed efficiencies and healthy food supplies^5^. In addition to its applications in animal agriculture, *MSTN* is also directly or indirectly involved in the regulation of fat and glucose metabolism^6^,^7^. These results suggest that the inhibition of *MSTN* function can potentially be used as a treatment for obesity and diabetes. It is possible that selective breeding for specific *MSTN* mutations might result in increased muscle mass and greater commercial value in Small Tailed Han sheep (STH sheep).

Sheep and goats serve as particularly good animal models due to their appropriate body size and easy management^8^. STH sheep (*Ovis aries*) are a meat and hair breed originating from Mongolian sheep in ancient northern China, but the breed has a slow growth rate and poor feed efficiency^9^,^10^. These unique qualities make STH sheep a suitable model to test the effects of *MSTN* mutations on muscle growth. In addition, the silencing of this gene in breeds that are specialized for the production of superfine or ultrafine wool could be an interesting model for producing more meat in a high quality wool producing animal.

Recent advancements in genetic manipulation techniques have made it possible to successfully target a gene with a high efficiency^11^,^12^. The direct modification of zygotic genomes using zinc-finger nuclease and CRISPR/Cas9 technology has been used to generate gene-edited sheep and goats^13^,^14^,^15^. Although the direct modification of zygotic genomes may have some advantages, including convenient gene manipulation, this strategy may result in mosaic or hypomorphic mutations^16^,^17^,^18^,^19^. In such cases, targeted mutations may not be transmitted to offspring^20^, and one or two more rounds of breeding may be required to obtain homozygous animals^16^. In contrast, somatic cell gene editing followed by somatic cell nuclear transfer (SCNT) permits the screening of appropriate mutant cells before animal production and ensures that the animals harbor the expected gene modifications^17^ or the precise allele replacements at the cellular level. Gene editing at the cellular level followed by SCNT has been successfully implemented in several species^17^,^18^,^19^,^21^,^22^,^23^ but not in sheep. Using the advantages of transcription activator-like effector nuclease (TALEN) technology, we attempted to disrupt the *MSTN* gene in the somatic cells of STH sheep by combining TALEN-mediated gene modification with SCNT.

In this study, we generated genetically modified sheep via gene editing in the somatic cells of STH sheep using TALEN technology followed by SCNT to produce *MSTN*-knockout (KO) STH sheep. Phenotypic analyses and functional assays of mutated sheep were also performed. The generation of *MSTN*-null sheep provides a genetic improvement in sheep breeds for meat production and an important large-animal model for biomedical research on musculoskeletal formation, development, and diseases.

## Results

### Generation and identification of *MSTN*-KO sheep

**Figure 1:**
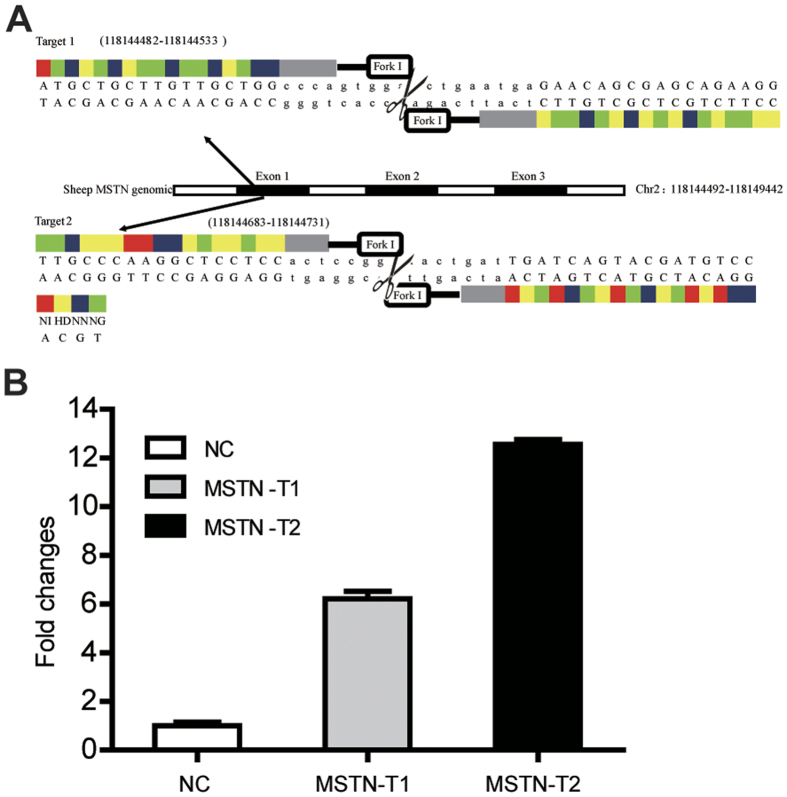
TALEN design and activity. (**A**) A schematic of TALEN targeting of the ovine *MSTN* locus depicting exon 1 of the ovine *MSTN* gene and the designed target 1 and target 2. (**B**) The detection of TALEN activity using a luciferase SSA recombination assay. Luciferase activity was increased by 6.2- and 12.6-fold for *MSTN*-T1 and *MSTN*-T2, respectively, compared with the control activity.

We established a STH sheep fetal fibroblast cell line as described in the methods section. The construction of the TALENs are shown in Fig. 1A. TALEN activity was tested, and we found that luciferase activity was increased by 6.2- and 12.6-fold by *MSTN*-T1 and *MSTN*-T2, respectively, compared with the activity of the control (Fig. 1B). *MSTN*-T1 was transfected into fetal fibroblast cells (ST1), and 212 single cell-derived cell colonies were obtained and identified via gene sequencing. The rate of monoallelic KO was 6.6% (14/212), and no biallelic mutations were detected (Tables 1 and 2). Similarly, *MSTN*-T2 transfection resulted in 111 single cell-derived cell colonies. The rate of mutation was 9.9% (11/111), with monoallelic and biallelic *MSTN*-KO rates of 4.5% (5/111) and 5.4% (6/111), respectively (Tables 1 and 3). Meanwhile, we confirmed that the cell colonies harbored the MSTN gene mutation in MSTN-T1 and MSTN-T2 via PCR and a T7 endonuclease I assay (Fig. S1). Among these colonies, the cell clone ST2-22 derived from MSTN-T2 transfected cells contained a biallelic KO consisting of ACT and GGAA deletions, resulting in a loss-of-function mutation of the *MSTN* gene. Thus, we selected the cell clone ST2-22 as the nuclear donor for the SCNT.

**Figure 2:**
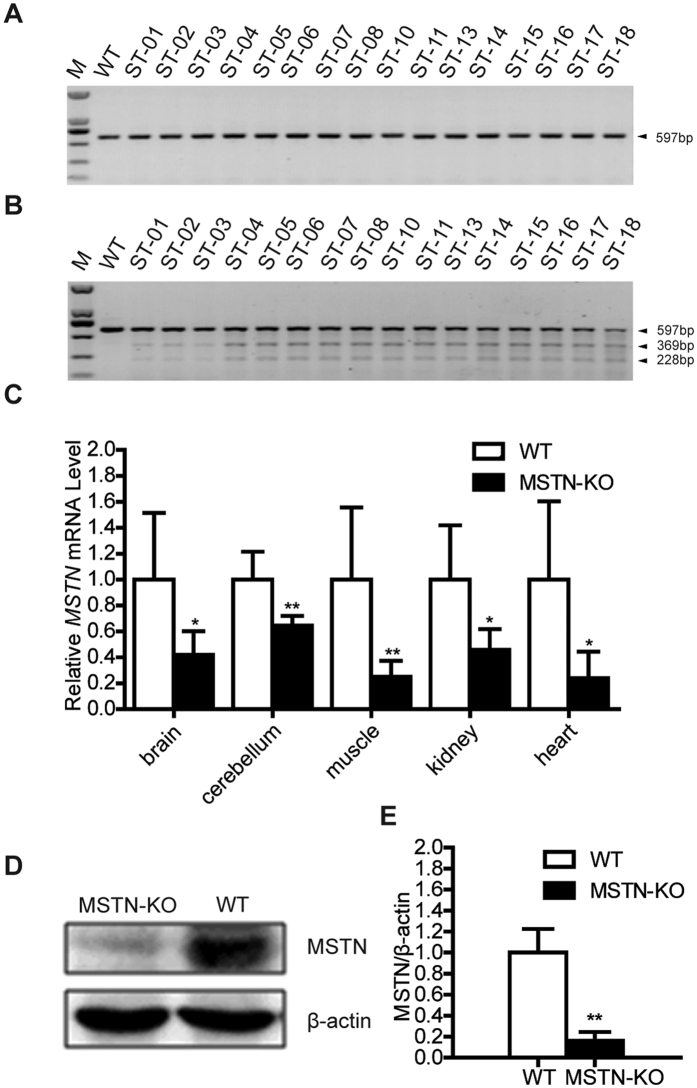
Identification of transgenic lambs. (**A**) The detection of the MSTN gene in lambs via PCR. The genomic regions surrounding the target site were amplified, and a 597 base pair PCR product of the MSTN gene was obtained. Analyses of wild-type (WT) lamb and lamb ST-01 to ST-18 genomic regions are shown. (**B**) Genotyping of MSTN mutant lambs using the T7 endonuclease I assay. MSTN genes of each lamb were assayed and are presented in the same order as the PCR results. Samples showing one band indicate the WT allele, while mutated alleles produced three bands in a Surveyor endonuclease assay. (**C**) The relative expression levels of *MSTN* mRNA in the different tissues from MSTN-KO and WT sheep. The relative expression levels of *MSTN* mRNA in brain, cerebellum, muscle, kidney, heart, liver and kidney tissues of MSTN-KO and WT sheep were measured via q-PCR. But only five tissues have detectable expression of *MSTN* and were showed. Expression of the GAPDH gene was used to normalize the values of MSTN. *p < 0.05 and **p  < 0.01 denote significant differences in MSTN-KO lambs compared with WT lambs. (**D**) Protein expression levels were assessed via Western blotting. Myostatin protein expression in the muscle tissue of MSTN-KO and WT sheep are shown in cropped blots using an anti-MSTN monoclonal antibody. Anti-β-actin served as a loading control. (**E**) Quantification of relative MSTN protein levels. The staining intensities of the bands for MSTN and β-actin were quantified using Bio-Rad Image Lab software. Protein levels of MSTN were normalized to β-actin protein levels. **p  < 0.01 denotes a significant difference in *MSTN*-KO lambs compared with WT lambs.

The *in vitro* maturation (IVM) rate in oocytes was 58.4% (320/548). The cleavage and blastocyst rates were 82.3% (232/282) and 16.7% (47/282), respectively (Table 4). Reconstructed embryos generated via SCNT were transplanted into 37 naturally cycling females, 75.7% (28/37) of which became pregnant, and 15 recipients delivered. A total of 23 lambs were obtained, including 12 live lambs and 11 dead lambs (Table 4). We confirmed that 16 of the lambs born harbored the expected biallelic mutations of the MSTN gene via PCR (Fig. 2A), a T7 endonuclease I assay (Fig. 2B) and sequencing assays (Table 5), which indicated genotypes consistent with that of the nuclear donor cell.

To assess the specificity of TALEN cleavage, we identified the 100 most likely off-target sites (Supplementary Table S1) based on the sheep reference genome using the local-mixture model method TALENoffer^24^. We performed high-coverage whole-genome resequencing of an *MSTN* mutant genome and called confident small indels and single-nucleotide variants (SNVs) (see Methods). Our resequencing data confirmed the biallelic disruption of the *MSTN* target site (Supplementary Fig. S1), but no functional disruptions in the 100 predicted off-target sites were observed. Among the 100 off-target sites, 33 sites harbored SNPs, 2 sites had small indels, and 4 sites had both SNPs and small indels. All SNVs and indels were located in intergenic regions and introns, and thus were assumed to be non-functional (Supplementary Table S2). These results suggested the observed phenotypes are most possibly caused by target genes disruption rather than off targeting.

We used quantitative PCR (q-PCR) to analyze the expression levels of *MSTN* mRNA in five tissues from *MSTN*-KO and wild-type (WT) lambs. The results show that the mRNA levels in *MSTN*-KO tissue samples were significantly lower compared with levels in WT samples (*p* < 0.05) (Fig. 2C). Western blotting for *MSTN* revealed undetectable protein levels in *MSTN*-KO lambs but demonstrated the presence of the protein in WT lambs (Fig. 2D,E). These results suggest that the *MSTN* gene had been successfully knocked out in the experimental STH sheep.

### mRNA levels of *MSTN* signaling pathway-related genes

**Figure 3:**
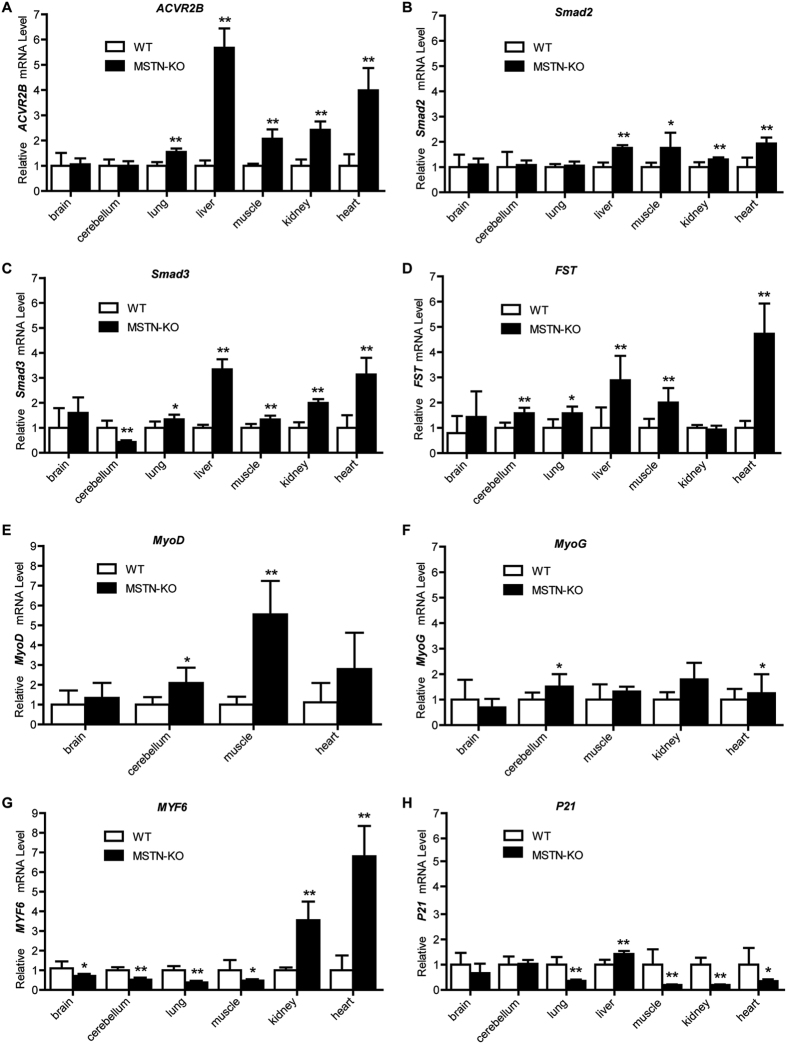
The relative expression levels of MSTN signaling pathway-related genes in the different tissues of MSTN-KO and WT sheep. The relative expression levels of (**A**) *ACV2B*, (**B**) *Smad2*, (**C**) *Smad3*, (**D**) *follistatin,* (**E**) *MyoD*, (**F**) *MyoG*, (**G**) *MYF6*, and (**H**) *P21* mRNA in brain, cerebellum, lung, liver, muscle, kidney and heart tissues of MSTN-KO and WT sheep were measured via q-PCR. But only 4, 5 and 6 tissues have detectable expression of *MyoD, MyoG and MYF6,* and were showed in (**E–G**). The expression of the *GAPDH* gene was used to normalize the values of the targeted genes. *p < 0.05 and **p < 0.01 denote significant differences in *MSTN*-KO lambs compared with WT lambs.

Several studies have indicated that MSTN gene KO can alter various MSTN signaling pathway-related factors, including myogenic regulatory factors (MRFs) (MYOD, MYOG, and MYF5), downstream signaling mediators (SMAD2 and SMAD3), the cell cycle regulator p21, the MSTN receptor ACVR2B and the MSTN antagonist follistatin (FST)^4^,^25^,^26^,^27^. Therefore, we further investigated the effect of MSTN KO on the mRNA expression of ACVR2B, Smad2, Smad3, FST, MYF6, MyoD, MyoG and P21 in different tissues via q-PCR. As expected, MSTN KO resulted in the up-regulation of FST, MyoD and MyoG, whereas the expression of P21 was down-regulated (*p* < 0.05), except in liver tissue. In addition, ACVR2B, Smad2 and Smad3 (except in cerebellum) expression levels were increased (*p* < 0.05). MYF6 expression was decreased in the brain, cerebellum, lung and muscle, whereas it was remarkably increased in the kidneys and heart tissues compared with the levels in WT lambs (*p* < 0.05, Fig. 3).

### Growth curve of *MSTN*-KO sheep

**Figure 4:**
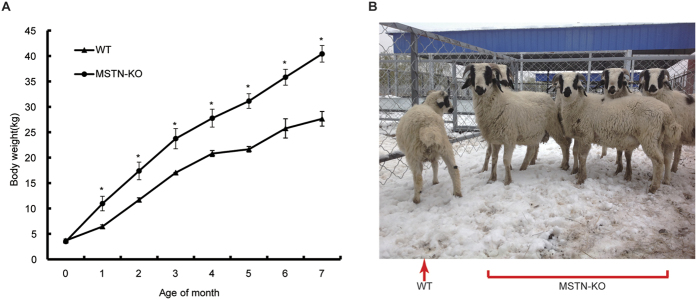
Characterization of the effects of *MSTN* gene KO in cloned sheep. (**A**) Changes in the average body weight of *MSTN*-KO lambs (n = 7) and WT lambs (n = 3) from birth to 7 month of age. Significant differences (*p < 0.05) during the 7 months after birth were found between *MSTN*-KO lambs and WT lambs. (**B**) Photos of WT and *MSTN*-KO sheep.

We measured the birth weight of *MSTN*-KO and WT newborn lambs, and no significant differences were found between the groups for live lambs (*p* > 0.05, Table 6). The growth curve of *MSTN*-KO and WT lambs during the 7 months after birth were recorded and revealed that the body weight of *MSTN*-KO sheep increased markedly faster than that of the WT lambs (*p* < 0.05, Fig. 4).

### Histological analysis

**Figure 5:**
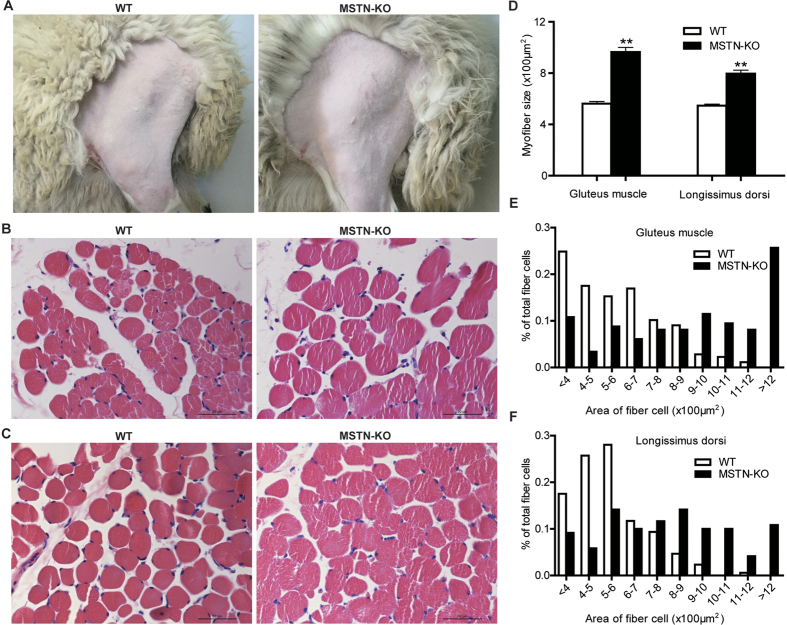
Histological analysis. (**A**) Photos of the gluteus muscles of WT and MSTN-KO sheep. (**B**) Hematoxylin and eosin-stained cross sections of the gluteus muscles. (**C**) Hematoxylin and eosin-stained cross sections of the longissimus dorsi muscles. Samples in panels A, B and C are presented in the same order. (**D**) Average size and density of myofibers in the gluteus and longissimus dorsi muscles. The relative size of myofibers in the gluteus from WT sheep (n = 177) and MSTN-KO sheep (n = 148) and the longissimus dorsi from WT sheep (n = 171) and MSTN-KO sheep (n = 120). *p < 0.05 and **p < 0.01 denote significant differences. (**E**) Distribution of different sizes of myofibers in gluteus muscles from WT sheep and *MSTN*-KO sheep. Samples were collected from 7-month-old sheep. (**F**) Distribution of different sizes of myofibers in the longissimus dorsi from a WT sheep and a *MSTN*-KO sheep. Samples were collected from 7-month-old sheep.

*MSTN*-KO sheep exhibited the double-muscled phenotype (Fig. 5A), and histological examination of the gluteus and longissimus dorsi showed muscle fiber hypertrophy relative to the fibers of the WT sheep (Fig. 5B,C). The average size of myofibers in the gluteus from *MSTN*-KO sheep (964.8 ± 439.6 μm^2^) was significantly larger than those of WT sheep (562.2 ± 219.9 μm^2^, *p* < 0.01). Similarly, the average size of myofibers in the longissimus dorsi from *MSTN*-KO sheep (796.2 ± 301.7 μm^2^) was substantially increased relative to those of WT sheep (546.2 ± 163.0 μm^2^, *p* < 0.01, Fig. 5D). The distribution of different sizes of gluteus and longissimus dorsi myofibers indicates that the percentage of smaller fiber cells in *MSTN*-KO sheep was lower than the percentage in WT sheep (Fig. 5E,F).

## Discussion

Gene editing at the cellular level enables the precise generation of animals with targeted gene modifications while avoiding the mosaicism that accompanies the direct microinjection of fertilized oocytes^17^. Recently, TALENs have been recognized as efficient gene editing tools and have been used in numerous experimental animals^28^. In this study, we generated MSTN biallelic-KO sheep via gene editing in somatic cells of STH sheep using TALEN technology followed by SCNT to produce MSTN-KO STH sheep. Genetically modified cattle have been created using TALENs and SCNT at the bovine albumin (bA) locus with a blastocyst rate of 9.8%^23^, and modified handmade cloning (HMC) methods have been used to produce transgenic sheep with a blastocyst rate of 8.9%^29^. In our study, the cleavage and blastocyst development rates were 82.3% and 16.7%, respectively. In addition, the cloning efficiency, obtained based on the total number of live lambs divided by the total number of recipients was 0.32 (12/37, Table 4). The production efficiency of mutant sheep was remarkably high. When we transplanted the embryos, we prepared for the activation of cloned embryos following 2 h, 24 h, and 48 h of culturing *in vitro*. According to the follicular development and ovulation times of the surrogates, we chose cloned embryos at different developmental stages, as well as different numbers of transplanted embryos. Sometimes, we mixed cloned embryos at two developmental stages for transplantation. We think that this approach may also be an effective strategy for improving cloning efficiency in sheep.

MSTN negatively regulates the development of skeletal muscle and growth. The increase in muscle size in MSTN-KO animals compared to that in WT animals has been shown to be due to fiber hyperplasia or the hypertrophy of skeletal muscle fibers in mice^1^, pigs^29^, cattle^30^, sheep^31^, and dogs^32^. In our study, the hypertrophy of skeletal muscle fibers was observed in MSTN-KO sheep (Fig. 5), which is consistent with the observation in the animals as mentioned above. MSTN is an essential regulator of the proliferation and differentiation of muscle cells during muscle development. Studies on muscle development have demonstrated that muscle fiber number is primarily determined before birth, and the diameter of myofibers expands after birth^33^,^34^,^35^. The different patterns of myofibers observed in WT and MSTN-KO individuals are likely related to postnatal muscle hypertrophy in the MSTN-KO sheep. The loss of MSTN functions can lead to an increase in the diameter of myofibers after birth^36^. During postnatal development, the diameter of myofibers in MSTN-KO sheep increased to a greater degree than was observed in WT sheep. MSTN knock down in transgenic sheep tends to result in faster growth rates than those observed in WT sheep^37^. In accordance with these results, we also found MSTN-KO sheep showed a tendency for faster growth rates than were observed in WT sheep (Fig. 4). These results suggest that MSTN KO possibly results in the hypertrophy of sheep myofibers. However, because our morphometric analysis was of only one muscle biopsy from *MSTN*-KO sheep and WT sheep, further research is required to clarify whether MSTN-knockdown in sheep causes myofiber hyperplasia or hypertrophy and how the effects may differ between fiber types.

MSTN exerts its effect via signaling though the cell surface receptor activin type IIB receptor (ACVR2B) and a Smad signaling pathway^4^,^38^,^39^. Smad2 and Smad3 are the transcription factors downstream of myostatin and can induce atrophy^40^. Follistatin (FST) has been shown to bind to some TGF-β family members and can function as a potent myostatin antagonist. The overexpression of follistatin as a result of transgenic modifications in muscle has been shown to increase muscle growth *in vivo*^4^, and a lack of follistatin results in reduced muscle mass at birth^41^. Furthermore, the increased muscle mass in MSTN-null mice and transgenic mice expressing high levels of the follistatin or a dominant negative form of activin receptor type IIB (ActR IIB) has been shown to result from both myofiber hyperplasia and hypertrophy^4^,^42^,^43^. In according with these results, we also found that the expression levels of *ACV2B*, *Smad2*, *Smad3* and *FST* in muscle, lung, liver and heart tissues were significantly increased following MSTN KO (Fig. 3A–D). MRFs, including *MyoD*, *MRF4*, and *MyoG,* play a critical role in myogenic differentiation^44^,^45^,^46^. The expression levels of the *MyoD* and *MyoG* genes are negatively regulated by the *MSTN* gene, and therefore, these MRFs are up-regulated in *MSTN*-null (−/−) mouse muscle tissue^26^. Studies on knockout mice have also shown a relationship between different MRFs in which the absence of one is compensated for by another^47^,^48^. Our observations of *MyoD* and *MyoG* expression levels in KO sheep are in agreement with these results (Fig. 3E,F). However, our findings show that *MYF6* expression was decreased in brain, cerebellum, lung and muscle tissues, whereas it was remarkably increased in kidney and heart tissues compared with its levels in WT lambs (Fig. 3G). As reported earlier, MSTN enhances the expression of the cell cycle inhibitor p21 leading to the negative regulation of cell proliferation^27^,^49^. In accordance with previous studies, MSTN KO resulted in the downregulation of *p21* in sheep muscle, kidney, heart and lung tissues (Fig. 3H). Thus, the increase in *MyoD*, *MyoG*, *ACV2B*, *Smad2*, *Smad3* and *follistatin,* as well as the decrease in *P21,* caused by MSTN KO may also result in the ability of cells to properly exit the cell cycle, leading to increased myogenic differentiation. Further study is needed to determine the detailed mechanisms underlying the regulation of the genes described above in response to *MSTN* KO at different development stages of sheep, as well as at the cellular level.

In conclusion, we have successfully generated *MSTN* mutant STH sheep via gene editing in somatic cells using TALEN technology in combination with SCNT. *MSTN*-KO sheep developed and grew normally and exhibited increased body weight and muscle growth relative to WT sheep. The generation of *MSTN*-null sheep could provide genetic improvements to sheep breeds for meat production and could serve as an important large-animal model for biomedical research on diseases.

## Materials and Methods

### Animal care and the establishment of Small Tail Han sheep fetal fibroblast cells

Animal use and care were in accordance with animal care guidelines that conformed to the Guide for the Care and Use of Laboratory Animals published by the US National Institutes of Health (NIH Publication No. 85-23). All animal experiments were performed with the approval of the Animal Care and Use Committee of Yunnan Agricultural University. In this study, we selected Small Tail Han (STH) sheep as our research subject and established a sheep fetal fibroblast cell line. Fibroblast cells were isolated from a 35-day-old fetus (♂). The fetal tissues were washed three times in sterile phosphate-buffered saline (PBS) containing 5% penicillin and streptomycin (PS) and were then washed an additional five times with PBS. The tissues were cut into small pieces and transferred into a T25 culture flask. Then, 4 mL collagenase IV was added, and the tissues were digested on a horizontal shaker in an incubator at 37 °C for 4 h. Following the removal of collagenase via centrifugation, the collected cells were cultured at 37 °C in a 5% CO_2_ incubator with Dulbecco’s modified Eagle’s medium (DMEM) culture medium containing 10% fetal bovine serum (FBS) and 1% PS. When fibroblast cells reached 80% confluence, they were frozen and stored in liquid nitrogen for future use.

### Construction and testing of the gene editing plasmids

TALENs targeting exon 1 of the sheep MSTN gene (MSTN-T1 and MSTN-T2) were designed and assembled by ViewSolid Biotech (Beijing, China) (Fig. 1A). The TALEN target sites for MSTN-T1 and MSTN-T2 are provided in Supplementary Table S3. The *in vitro* activities of the TALEN plasmids were detected using a luciferase single strand annealing (SSA) recombination assay^48^. The assembled TALEN expression plasmids, SSA reporter plasmids, and Renilla plasmids were co-transfected into HEK293T cells using Lipofectamine TM 2000 (Invitrogen, USA). After 24 h, the cells were harvested and lysed in Luciferase Cell Lysis Buffer (Promega, USA). The relative luciferase activity was detected using a Dual-Luciferase Assay System (Promega, USA) and was measured using a SYNERGYMx Luminescence Microplate Reader (BioTek, USA). This experiment was repeated in triplicate.

### Establishment of *MSTN-*knockout (KO) cells

Prior to transfection, fetal fibroblast cells were thawed and cultured in DMEM (10% FBS, 1% PS) until subconfluence was reached. Approximately 7 × 10^5^ cells suspended in electro-transfection buffer were mixed with 10.5 μg of the TALEN plasmid pair (*MSTN*-T1 and *MSTN*-T2) in a final sample volume of 700 μL. The cell suspension was loaded into a 4 mm gap cuvette and subjected to an electrical treatment of one pulse at 250 V for 25 ms (Bio-Rad Gene Pulser Xcell, USA). Then, the electro-transfection buffer was decanted, and the cells were seeded in 5 mL of fresh DMEM containing 10% FBS in a T25 culture flask following a 48 h incubation at 37 °C. The cells were then detached via trypsinization, and the extremely dilute culture method was used to cultivate the cells. We eventually obtained a 100 μL cell suspension of approximately 100 cells. The cells were then cultured in 10 cm diameter dishes. After 12 d, the colonies were assessed via polymerase chain reaction (PCR) (upstream primer, 5′-TGTCTCTCAGACTGGGCAGGC-3′; downstream primer, 5′-CCTTACGTACAAGCCAGCAGC-3′), and the amplified fragments were sequenced. We selected positive fibroblast cell lines with a biallelic KO as nuclear donors for SCNT.

### Oocyte collection and *in vitro* maturation (IVM)

Ovaries were collected from sheep from two abattoirs (Inner Mongolia Grassland HongBao Food Co., TD; Inner Mongolia Mei Yang Yang Food Co., TD) and were transported to the laboratory in a thermostatic container in 0.9% (w/v) NaCl solution at approximately 37 °C. Oocyte collection and *in vitro* maturation were performed as described previously^50^. Briefly, cumulus-oocyte complexes (COCs) were obtained from follicles with a diameter of 2 to 6 mm. Oocytes surrounded by a minimum of three cumulus cell layers were selected and cultured for 20 to 22 h in Medium 199 containing 10% (v/v) FBS, 10 μg/mL Fsh, 10 μg/mL LH, 0.1 mg/mL l-cysteine hydrochloride monohydrate, 10 ng/mL epidermal growth factor, 1 μg/mL 17-β-estradiol and 75 mg/mL potassium penicillin G at 38.5 °C in 100% humidity with 5% CO_2_.

### Somatic cell nuclear transfer

SCNT was performed as described previously with slight modifications^50^. The COCs were cultured in maturation medium at 38.5 °C in a humidified atmosphere for 22–24 h. Cumulus cells of the COCs were removed by exposure to Medium199 containing 0.1% hyaluronidase. Oocytes extruding the first polar body with uniform cytoplasm were selected, and enucleation was performed using a 20 μm diameter pipette by aspirating the first polar body and the surrounding cytoplasm in Medium199 containing 5 μg/mL cytochalasin B (CB) and 10% FBS. MSTN-KO fibroblast cells were used as nuclear donors. A single donor cell nucleus was injected into the perivitelline space of an enucleated oocyte to form the donor cell-oocyte complexes. The reconstructed embryos were fused using an Electro Cell Fusion Generator LF201 (NEPA GENE Co., Ltd., Japan) with a double direct-current (DC) (150 V/mm, 20 μs, 1 s apart). Fused, reconstructed embryos were cultured for 1.5 h in G1 medium (Vitrolife, Sweden) and then were activated in 2.5 μM ionomycin for 5 min, followed by exposure to 2.0 mM 6-dimethyl aminopurine (DMAP) in G1 medium for 4 h. Following activation, embryos were transferred and cultured in G1 medium for 48–72 h. Then, the SCNT embryos were cultured in G2 medium (Vitrolife, Sweden) for 120 h under the same culturing conditions described above. Cleavage and blastocyst rates were calculated after 48 h and 168 h, respectively.

### Embryo transfer and the generation of *MSTN*-KO sheep

The SCNT embryos were cultured for 2 to 48 h and were surgically transferred into the oviducts of recipient sheep. Pregnancy was diagnosed after 50 d. When lambs were born, we collected ear tissues and extracted total DNA using a Tissue DNA Kit (OMEGA, D3396-2). We then assessed the *MSTN* gene via PCR (upstream primer, 5′-TGTCTCTCAGACTGGGCAGGC-3′; downstream primer, 5′-CCTTACGTACAAGCCAGCAGC-3′), T7 endonuclease I digestion and sequencing. The body weight and growth status of *MSTN*-KO lambs were recorded and compared with those of wild-type (WT) lambs.

### Off-target analysis

To exclude the possibility of off-target effects, we sequenced the genome of a randomly selected lamb using an Illumina 2500 sequencer. We obtained 108 Gb of high quality reads using paired-end 150 bp (PE150) sequencing, amounting to 40X coverage of the sheep reference genome^51^. In total, 98.02% of the reads were aligned to the sheep reference genome using BWA MEM software (version 0.7.12-r1039)^49^ with the following parameters: -k17 -B 3 -O 5, 5 -t 5 -r 3. We then sorted the aligned bam file using Picard (version V1.84) (http://sourceforge.net/projects/picard/) and realigned the indels using RealignerTargetCreator and IndelRealigner in GATK (version 3.3)^52^. We called small indels and single-nucleotide variants (SNVs) using samtools mpileup (version 1.2)^53^ with the default parameters. Finally, false positive SNPs and indels were filtered using BCFtools (BCFtools view) with the following parameters: -I ‘(TYPE = “indel” | TYPE = “snp”) & MIN (DP) > 5 & MIN (MQ > 20) & MAX (DP) 50’. After obtaining definitive indels, we identified possible sites of off-target TALEN activity using a local-mixture model that models binding specificity and independently takes into account the importance of repeat-variable di-residues (RVDs)^5^. We used the first RVD sequence (NI-NG-NN-HD-NG-NN-HD-NG-NG-NN-NG-NG-NN-HD-NG-NN-NN), the second RVD sequence (HD-HD-NG-NG-HD-NG-NN-HD-NG-HD-NN-HD-NG-NN-NG-NG-HD), and a 12 to 24 space length as parameters for predicting off-target sites.

### RNA isolation and qPCR

Two *MSTN*-KO lambs and two age-matched WT lambs were sacrificed 2 days after birth. Various tissues, including brain, cerebellum, lung, liver, muscle, kidney and heart, were obtained. Total RNA was isolated using TRIzol (Invitrogen, USA) according to the manufacturer’s instructions. cDNA was synthesized from total RNA using a PrimeScript RT reagent Kit (TAKARA, Japan). The obtained cDNA was used as a template in SYRB green-based q-PCR (CFX-96, Bio-Rad, USA). The primers can be found as Supplementary Table S4. The mRNA expression levels of the *MSTN*, *ACVR2B*, *Smad2*, *Smad3*, *FST*, *MYF6*, *MyoD*, *MyoG* and *P21* were assessed by Quantitative-polymerase chain reaction (q-PCR). *GAPDH* was used for normalization.

### Protein extraction and immunoblotting

We selected the muscle tissues described above, detected the protein of MSTN by western blotting compared with WT lambs. Muscle tissues were lysed in RIPA lysis buffer (Beyotime, China) with protease inhibitors at 4 °C. After lysis, supernatants were obtained by centrifugation at 14,000 × g for 15 min at 4 °C. The protein (50 μg) were separated using SDS-PAGE. After electrophoresis, the proteins were transferred to PVDF membrane and reacted with primary antibodies against MSTN (anti-MSTN, 1:1000, Thermo Scientific) and β-actin (anti-β-actin, 1:5000, Sigma-Alderich) at 4 °C overnight. After incubation, membranes were washed and incubated with anti-mouse or anti-rabbit secondary antibodies (R&D, USA). The membranes were developed using the ECL detection system (Easysee Western Blot Kit, China) and visualized with an Imagining System (Bio-Rad, Universal Hood II, USA).

### Histological analysis

The muscle samples of a MSTN-KO and a WT sheep of the same age and location were collected for histochemistry. Each tissue was fixed in paraformaldehyde (4%), dehydrated in a graded alcohol series, and then embedded in paraffin. Paraffin-embedded tissues were sectioned at 3–5 μm. The slices were then stained with hematoxylin and eosin (HE). Slides were viewed via microscopy (Leica, DM2000, Germany). For each sample, 5 fields of view (areas) were randomly selected in HE stained sections using a 40x objective and then analyzed using Leica LAS Core software. For each sample area, 100–200 myofibers were measured, and the relative size of myofibers and the distribution of different sizes of myofibers were determined.

### Statistical analysis

Statistical comparisons of birth weight, the relative mRNA level of the genes, the protein expression level of *MSTN* and myofiber size between wild-type and MSTNKO sheep were performed by the Student’s *t*-test and *p* < 0.05 was considered as statistically significant. Statistical analyses were carried out using SPSS 22.0 software package (IBM Corp, Armonk, NY).

## Additional Information

**How to cite this article**: Li, H. *et al.* Generation of biallelic knock-out sheep via gene-editing and somatic cell nuclear transfer. *Sci. Rep.* **6**, 33675; doi: 10.1038/srep33675 (2016).

## Supplementary Material

Supplementary Information

## Figures and Tables

**Table 1:** Targeting efficiency of TALEN Set#1 and Set#2.

Target	No. colonies	Monoallelic-KO (%)	Biallelic-KO (%)
TALEN Set#1	212	14 (6.6)	—
TALEN Set#2	111	5 (4.5)	6 (5.4)

**Table 2:** TALEN-mediated mutations in the fetal fibroblast cells of ST1.

Number	Sequence	Deletion (Δ), insertion (+) or point mutation (p) NO. of base pairs
WT	5′–CCCAGTGGATCTGAATGA–3′	
ST1-39	5′–CCCAGTGGATCTGAATGA–3′/5′–CCCAG- - - - - - TGAATGA–3′	WT/Δ6
ST1-49	5′–CCCAGTGGATCTGAATGA–3′/5′- - - - - - - - - - - - - - - - - - - - - -- - - - - GAATGA–3′	WT/Δ27
ST1-64	5′–CCCAGTGGATCTGAATGA–3′/5′–CCCAG - - - - - - - - - - TGA–3′	WT/Δ10
ST1-86	5′–CCCAGTGGATCTGAATGA–3′/5′–- - - - - - - - - - - - GAATGA(CAGCAACAGAAGG)- - - - - - - - - - - - - - -–3′	WT/+13Δ27
ST1-106	5′–CCCAGTGGATCTGAATGACCCAG –3′/5′–- - - - - - - - - -TGA–3′	WT/Δ10
ST1-121	5′–CCCAGTGGATCTGAATGA–3′/5′–- - - - - - - - - - - - - - ATGA–3′	WT/Δ14
ST1-126	5′–CCCAGTGGATCTGAATGA–3′/5′–CCCAGTGttgtgGAATGA–3′	WT/p5
ST1-138	5′–CCCAGTGGATCTGAATGA–3′/5′–CCCAGc- - - - - - - - -TGA–3′	WT/p1Δ9
ST1-195	5′–CCCAGTGGATCTGAATGA–3′/5′–CCCAG- - GAT- - - AATGA–3′	WT/Δ5
ST1-200	5′–CCCAGTGGATCTGAATGA–3′/5′–CCCA- - - - - - - - GAATGA–3′	WT/Δ8
ST1-207	5′–CCCAGTGGATCTGAATGA–3′/5′–CCCAG- - - - - - TGAATGA–3′	WT/Δ6
ST1-215	5′–CCCAGTGGATCTGAATGA–3′/5′–- - - - - - - - - - - - - - - - - - - - -–3′	WT/Δ21
ST1-221	5′–CCCAGTGGATCTGAATGA–3′/5′–CCCAG- - - - - - TGAATGA–3′	WT/Δ6
ST1-225	5′–CCCAGTGGATCTGAATGA–3′/5′–······–3′	WT/Δ67

WT sequence is shown above. Deletion, insertion and point mutation (denoted with “Δ” , “+” and “p” with the number of base pairs) are identified.

**Table 3:** TALEN-mediated mutations in the fetal fibroblast cells of ST2.

Number	Sequence	Deletion (Δ), insertion (+) or point mutation (p) NO. of base pairs
WT	5′–ACTCCGGGAACTGAT–3′	
ST2-22	5′–ACTCCGGGA - - - GAT–3′/5′–ACTCCG - - - -CTGAT–3′	Δ3/Δ4
ST2-24	5′–ACTCCG(T) - - - ACTGAT–3′/5′–ACTCCGGGA(AGGA)ACTGAT–3′	+1Δ3/+4
ST2-26	5′–ACTCCGGGAACTGAT–3′/5′–- - - - - - - - - ACTGAT–3′	WT/Δ9
ST2-43	5′–ACTCCGGGAACTGATACTCCG–3′/5′–- - - - CTGAT–3′	WT/Δ4
ST2-62	5′–- - - - - - - - - - - - - - - - - CTGAT–3′/5′–ACTCCGA- - - CTGAT–3′	Δ17/Δ3
ST2-63	5′–ACTCCGGGAACTGAT–3′/5′–- - - - - - - - - - - - - - - - - CTGAT–3′	WT/Δ17
ST2-72	5′–ACT - - - - - - - CTGAT–3′/5′–ACTCCGG- - - CTGAT–3′	Δ7/Δ3
ST2-75	5′–ACTCCGGGAACTGAT–3′/5′–ACTCCGGGA(GG)ACTGAT–3′	WT/+2
ST2-76	5′–ACTCCGGGAACTGAT–3′/5′–ACTCCG - - - - CTGAT–3′	WT/Δ4
ST2-89	5′–ACTaC - - - - - - - - - -–3′/5′–AC - - - - - - - ACTGAT–3′	p1Δ10/Δ7
ST2-102	5′–ACTCCG - - - - - - GAT–3′/5′–ACTC- - - - - - - TGAT–3′	Δ6/Δ7

WT sequence is shown above. Deletion, insertion and point mutation (denoted with “Δ” , “+” and “p” with the number of base pairs) are identified.

**Table 4:** Nuclear transfer efficiencies of SCNT.

Donor cells	No. of reconstructed embryos	Cleavage (%)	Blastocyst (%)	No. of recipients	No. of pregnancy (%)	No. of delivery (%)	Lambs (live)
ST2-22	282	232(82.3 ± 2.1)	47 (16.7 ± 3.8)	37	28 (75.7)	15 (40.5)	23 (12)

**Table 5:** TALEN-mediated mutations in the lambs.

Number	Live or death	Sequence	Deletion (Δ) or insertion (+) NO. of base pairs
WT		ACTCCGGGAACTGAT	
ST-01	Live	ACTCCGGGA- - - GAT	Δ3
		ACTCCG - - - - CTGAT	Δ4
ST-02	Live	ACTCCGGGA- - - GAT	Δ3
		ACTCCG - - - - CTGAT	Δ4
ST-03	Live	ACTCCGGGA- - - GAT	Δ3
		ACTCCG - - - - CTGAT	Δ4
ST-04	Death	ACTCCGGGA- - - GAT	Δ3
		ACTCCG - - - - CTGAT	Δ4
ST-05	Live	ACTCCGGGA- - - GAT	Δ3
		ACTCCG - - - - CTGAT	Δ4
ST-06	Live	ACTCCGGGA- - - GAT	Δ3
		ACTCCG - - - - CTGAT	Δ4
ST-07	Live	ACTCCGGGA- - - GAT	Δ3
		ACTCCG - - - - CTGAT	Δ4
ST-08	Live	ACTCCGGGA- - - GAT	Δ3
		ACTCCG - - - - CTGAT	Δ4
ST-10	Death	ACTCCGGGA- - - GAT	Δ3
		ACTCCG - - - - CTGAT	Δ4
ST-11	Death	ACTCCGGGA- - - GAT	Δ3
		ACTCCG - - - - CTGAT	Δ4
ST-13	Live	ACTCCGGGA- - - GAT	Δ3
		ACTCCG - - - - CTGAT	Δ4
ST-14	Live	ACTCCGGGA- - - GAT	Δ3
		ACTCCG - - - - CTGAT	Δ4
ST-15	Live	ACTCCGGGA- - - GAT	Δ3
		ACTCCG - - - - CTGAT	Δ4
ST-16	Live	ACTCCGGGA- - - GAT	Δ3
		ACTCCG - - - - CTGAT	Δ4
ST-17	Live	ACTCCGGGA- - - GAT	Δ3
		ACTCCG - - - - CTGAT	Δ4
ST-18	Death	ACTCCGGGA- - - GAT	Δ3
		ACTCCG - - - - CTGAT	Δ4

**Table 6:** Comparison of birth weight between MSTN-KO and WT lambs.

Type of lambs	*MSTN*-KO lambs (♂)			WT (♂)
	Total	Live	Dead	
No. of lambs	23	12 (52.2%)	11 (47.8%)	10
Birth weight (kg)	3.0 ± 0.3	3.5 ± 0.4	2.3 ± 0.5	3.6 ± 0.1

The WT group consisted of non-knockout *MSTN* gene lambs. Data for birth weight of ten wild type lambs was provided by Bayannaoer Livestock Improvement Station.

The birth weights are expressed as the mean ± SE.
