# Supplementary material for: Generation of biallelic knock-out sheep via gene-editing and somatic cell nuclear transfer
Source: Sci Rep. 2016 Sep 22;6:33675. doi: 10.1038/srep33675 (PMC5031972; doi:10.1038/srep33675)
Supplement: Supplementary Information [file srep33675-s1.pdf]

Supplementary information

Generation of biallelic knock-out sheep via gene-editing and somatic cell nuclear transfer

Honghui Li[1,2#], Gui Wang[3#], Zhiqiang Hao[4,5#], Guozhong Zhang[6,7], Yubo Qing[2,6], Shuanghui Liu[4], Lili Qing[6], Weirong Pan[2], Lei Chen[5], Guichun Liu[5], Ruoping Zhao[5], Baoyu Jia[2,6], Luyao Zeng[2,6], Jianxiong Guo[2,6], Lixiao Zhao[2,6], Heng Zhao[1,2], Chaoxiang Lv[1,6], Kaixiang Xu[1,6], Wenmin Cheng[2], Hushan Li[8], Hong-Ye Zhao[1,6*], Wen Wang[5*], Hong-Jiang Wei[1,6,7*]

[1]State Key Laboratory for Conservation and Utilization of Bio-Resources in Yunnan, Yunnan Agricultural University, Kunming 650201, China. [2]College of Animal Science and Technology, Yunnan Agricultural University, Kunming, China. [3]College of Hetao, Bayannaoer 015000, China. [4]Inner Mongolia Zhong-Ke-Zheng-Biao Biotech Co,.Ltd, Bayannaoer 015400, China. [5]State Key Laboratory of Genetic Resources and Evolution, Kunming Institute of Zoology, Chinese Academy of Sciences, Kunming 650223, China. [6]Reproductive & Developmental Laboratory, Southwest China Biodiversity Laboratory, Kunming 650203, China. [7]Key Laboratory Animal Nutrition and Feed of Yunnan Province, Yunnan Agricultural University, Kunming 650201, China. [8]Bayannaoer Livestock Improvement Station, Bayannaoer 015000, China.

*Corresponding Author e-mail：hongjiangwei@126.com

This file contains twelve pages with four table (Table S1-S4) and three figures (Figures S1-S3).

Table S1 The possible off-target sites.

<table>
<tr><th># ID</th><th>Position 1</th><th>Position 2</th><th>Distance</th><th>Sequence 1</th><th>Matches 1</th><th>Sequence 2</th><th>Matches 2</th><th>Architecture</th><th>Full site</th><th>Score</th></tr>
<tr><td>chr2</td><td>6313955</td><td>6313991</td><td>18</td><td>TATGCTGCTTGTTGCTGG</td><td>M||:||:|||:||:||::</td><td>TCCTTCTGCTCGCTGTTC</td><td>M||||||:|||:||:|||</td><td>first-second</td><td>TATGCTGCTTGTTGCTGGcccagtggatctgaatgaGAACAGCGAGCAGAAGGA</td><td>−0.49385249</td></tr>
<tr><td>chr2</td><td>21369577</td><td>21369608</td><td>13</td><td>TCCCTCTGCACGCTGCCT</td><td>M||x|||:|x|:||:xxx</td><td>TCCTTCTGTTCCCTGTTC</td><td>M||||||:x||x||:|||</td><td>second-second</td><td>TCCCTCTGCACGCTGCCTggcttccctcaggGAACAGGGAACAGAAGGA</td><td>−1.403578065</td></tr>
<tr><td>chr17</td><td>32490313</td><td>32490347</td><td>16</td><td>TCCTTCTAATCCCTGATC</td><td>M||||||xx||x||:x||</td><td>TATGCTGATTTATGCTGA</td><td>M||:||:x||xx|:||:x</td><td>second-first</td><td>TCCTTCTAATCCCTGATCaaaatttataattgatTCAGCATAAATCAGCATA</td><td>−1.430665566</td></tr>
<tr><td>chr20</td><td>8292741</td><td>8292779</td><td>20</td><td>TCCTCATGCTCTCTGCTC</td><td>M|||xx|:|||x||:x||</td><td>TCCTTCTGCTCCCTGTAG</td><td>M||||||:|||x||:|xx</td><td>second-second</td><td>TCCTCATGCTCTCTGCTCtttagggataagaaagccagCTACAGGGAGCAGAAGGA</td><td>−1.440607321</td></tr>
<tr><td>chr17</td><td>12117522</td><td>12117554</td><td>14</td><td>TAGGCTGATTCTTGCTGC</td><td>M|x:||:x||x||:||:x</td><td>TCTGCTGCATGTTGCTCA</td><td>Mx|:||:|x|:||:||xx</td><td>first-first</td><td>TAGGCTGATTCTTGCTGCgattcccatagaacTGAGCAACATGCAGCAGA</td><td>−1.448147365</td></tr>
<tr><td>chr2</td><td>6313955</td><td>6313993</td><td>20</td><td>TATGCTGCTTGTTGCTGG</td><td>M||:||:|||:||:||::</td><td>TTTCCTTCTGCTCGCTGT</td><td>Mx|x||x||xx|x:||:x</td><td>first-first</td><td>TATGCTGCTTGTTGCTGGcccagtggatctgaatgagaACAGCGAGCAGAAGGAAA</td><td>−1.459826979</td></tr>
<tr><td>chr20</td><td>8292741</td><td>8292775</td><td>16</td><td>TCCTCATGCTCTCTGCTC</td><td>M|||xx|:|||x||:x||</td><td>TCTGCTCCCTGTAGCTGG</td><td>Mx|:||x|x|:|x:||::</td><td>second-first</td><td>TCCTCATGCTCTCTGCTCtttagggataagaaagCCAGCTACAGGGAGCAGA</td><td>−1.479978149</td></tr>
<tr><td>chr14</td><td>78168541</td><td>78168581</td><td>22</td><td>TCCTCCCCTTTCCTGTTC</td><td>M|||x|xxx|xx||:|||</td><td>TCCTTCTGTTCTCTGTTC</td><td>M||||||:x||x||:|||</td><td>second-second</td><td>TCCTCCCCTTTCCTGTTCcgccagatctaaattggtcttgGAACAGAGAACAGAAGGA</td><td>−1.536749422</td></tr>
<tr><td>chr15</td><td>69632598</td><td>69632632</td><td>16</td><td>TATTCTTCTTGTAGCTGA</td><td>M||x||x|||:|x:||:x</td><td>TCTTTCTACTCACCCTTC</td><td>M|x|||x|||x|xx|||</td><td>first-second</td><td>TATTCTTCTTGTAGCTGAatctgaaagttctcagGAAGGGTGAGTAGAAAGA</td><td>−1.542134119</td></tr>
<tr><td>chr3</td><td>22313921</td><td>22313952</td><td>13</td><td>TATGCAGCTTGGTACTAG</td><td>M||:|x:|||:x|x||x:</td><td>TATCCTCCTTCTTGCTTT</td><td>M||x||x|||x||:||xx</td><td>first-first</td><td>TATGCAGCTTGGTACTAGgaaaggggaagcaAAAGCAAGAAGGAGGATA</td><td>−1.55419318</td></tr>
<tr><td>chr21</td><td>12050595</td><td>12050631</td><td>18</td><td>TCCTTCTGCGCCCTGTCC</td><td>M||||||:|x|x||:|x|</td><td>TCAGCTGCTTGCGGCAGG</td><td>Mxx:||:|||:xx:|x::</td><td>second-first</td><td>TCCTTCTGCGCCCTGTCCtgcctgtctccgctggcaCCTGCCGCAAGCAGCTGA</td><td>−1.574627167</td></tr>
<tr><td>chr26</td><td>28740108</td><td>28740150</td><td>24</td><td>TAAGCTCTTTCTTGCTGA</td><td>M|x:||xx||x||:||:x</td><td>TAAGCTGCTTTTCACTGG</td><td>M|x:||:|||x|xx||::</td><td>first-first</td><td>TAAGCTCTTTCTTGCTGAtggtggttgtcataaatgtatgctCCAGTGAAAAGCAGCTTA</td><td>−1.58601299</td></tr>
<tr><td>chr12</td><td>68932365</td><td>68932404</td><td>21</td><td>GCCTTCTGCTCACTGTTC</td><td>m||||||:|||x||:|||</td><td>CCATTCTCCCCCAAATTC</td><td>m|x|||x|x|xxxx|||</td><td>second-second</td><td>GCCTTCTGCTCACTGTTCtgttacagtttggatgggaggGAATTTGGGGGAGAATGG</td><td>−1.588332786</td></tr>
<tr><td>chr9</td><td>15855225</td><td>15855255</td><td>12</td><td>TAGACTGCTGGTTTCTGG</td><td>M|xx||:||x:||x||::</td><td>CCCACTACTTGTTGCTGG</td><td>mxxx||x|||:||:||::</td><td>first-first</td><td>TAGACTGCTGGTTTCTGGacatgggcatctCCAGCAACAAGTAGTGGG</td><td>−1.592173594</td></tr>
<tr><td>chr15</td><td>23913129</td><td>23913170</td><td>23</td><td>TCCCTCTCCTCCCCAGTC</td><td>M||x|||x|||x|xxx||</td><td>TCCTCCCCCTCCCTCTTC</td><td>M|||x|xx|||x||x|||</td><td>second-second</td><td>TCCCTCTCCTCCCCAGTCttaaccctgatagaatagatgcaGAAGAGGGAGGGGGAGGA</td><td>−1.597024312</td></tr>
<tr><td>chr1</td><td>133226599</td><td>133226633</td><td>16</td><td>CCCATCTACTCCCTGTTC</td><td>m||x|||x|||x||:|||</td><td>CCCTTCTCCTCTCTCTGA</td><td>m||||||x|||x||x|xx</td><td>second-second</td><td>CCCATCTACTCCCTGTTCttcagtttccccaacaTCAGAGAGAGGAGAAGGG</td><td>−1.597971371</td></tr>
<tr><td>chr13</td><td>17132488</td><td>17132528</td><td>22</td><td>CCCTTCTCCTGGCCCTTC</td><td>m||||||x||x:|xx|||</td><td>CCCCTCTCCTCGTTGTTC</td><td>m||x|||x|||:x|:|||</td><td>second-second</td><td>CCCTTCTCCTGGCCCTTCtcctaaggaggggctggggacaGAACAACGAGGAGAGGGG</td><td>−1.603010884</td></tr>
<tr><td>chr14</td><td>37026557</td><td>37026591</td><td>16</td><td>AGTCCTCCGTGTTGTTGG</td><td>mx|x||x|x|:||:x|::</td><td>TCTTTCTGCTCACTGTTC</td><td>M|x||||:|||x||:|||</td><td>first-second</td><td>AGTCCTCCGTGTTGTTGGggagaaggggtgtggaGAACAGTGAGCAGAAAGA</td><td>−1.60478917</td></tr>
<tr><td>chr17</td><td>53679578</td><td>53679619</td><td>23</td><td>TCTTCTGCTTATTTCTGG</td><td>Mx|x||:|||x||x||::</td><td>TATTTCTCCTTGCTATTC</td><td>Mxx||||x||x:||x|||</td><td>first-second</td><td>TCTTCTGCTTATTTCTGGaactctggaagcagagttccataGAATAGCAAGGAGAAATA</td><td>−1.606992233</td></tr>
<tr><td>chr6</td><td>35405629</td><td>35405665</td><td>18</td><td>TATGGTACATGTTACTAG</td><td>M||:x|x|x|:||x||x:</td><td>TCCTTCTGCCTCATGTTC</td><td>M||||||:|xxxx|:|||</td><td>first-second</td><td>TATGGTACATGTTACTAGcctcactttatgaataggGAACATGAGGCAGAAGGA</td><td>−1.608320424</td></tr>
<tr><td>chr3</td><td>39762888</td><td>39762922</td><td>16</td><td>TCATAATGCTCGCTCTTC</td><td>M|x|xx|:|||:||x|||</td><td>TCCTCTGCTTCTAGCTGG</td><td>Mxxx||:|||x|x:||::</td><td>second-first</td><td>TCATAATGCTCGCTCTTCtcccttcttgcactgaCCAGCTAGAAGCAGAGGA</td><td>−1.613594655</td></tr>
<tr><td>chr6</td><td>60529316</td><td>60529353</td><td>19</td><td>TCTACTGCCTGTCACTGG</td><td>Mx|x||:|x|:|xx||::</td><td>TAATTATGTTCACTGTTC</td><td>Mxx||x|:x||x||:|||</td><td>first-second</td><td>TCTACTGCCTGTCACTGGatgttttataaacaatgttGAACAGTGAACATAATTA</td><td>−1.624587321</td></tr>
<tr><td>chr11</td><td>86404351</td><td>86404388</td><td>19</td><td>TCCTTCTACTAGCTTCTC</td><td>M||||||x||x:||xx||</td><td>TAGGCTGCTTATCCCAGA</td><td>M|x:||:|||x|xx|x:x</td><td>second-first</td><td>TCCTTCTACTAGCTTCTCcgcagtgtttgttccaaatTCTGGGATAAGCAGCCTA</td><td>−1.63097977</td></tr>
<tr><td>chr7</td><td>99559623</td><td>99559657</td><td>16</td><td>GATACTGATTGTTGCTGC</td><td>m||x||:x||:||:||:x</td><td>TCTTTCTGCTCCCTCAAC</td><td>M|x||||:|||x||xxx|</td><td>first-second</td><td>GATACTGATTGTTGCTGCcctctgaatgcttacaGTTGAGGGAGCAGAAAGA</td><td>−1.633264073</td></tr>
<tr><td>chr26</td><td>28667329</td><td>28667364</td><td>17</td><td>TATGCTTCCTGTTACTGA</td><td>M||:||x|x|:||x||:x</td><td>TTGCCTGCTTGCTGCTTG</td><td>Mxxx||:|||:x|:||x:</td><td>first-first</td><td>TATGCTTCCTGTTACTGAgggcaggcataaacctcCAAGCAGCAAGCAGGCAA</td><td>−1.635364251</td></tr>
<tr><td>chr17</td><td>51217650</td><td>51217686</td><td>18</td><td>TCCTCCTCCTCCCTGTTC</td><td>M|||x||x|||x||:|||</td><td>CATCCTGCTGGGGTCTGG</td><td>m||x||:||x:xxx||::</td><td>second-first</td><td>TCCTCCTCCTCCCTGTTCtataaaagaacctggcatCCAGACCCCAGCAGGATG</td><td>−1.640719053</td></tr>
<tr><td>chr1</td><td>152375778</td><td>152375817</td><td>21</td><td>TCCTTCTCTTCCATATTC</td><td>M||||||xx||xx|x|||</td><td>TCCTCCTTCTCCCTGCTT</td><td>M|||x||x|||x||:x|x</td><td>second-second</td><td>TCCTTCTCTTCCATATTCccctgggaataccggaagcatAAGCAGGGAGAAGGAGGA</td><td>−1.643806223</td></tr>
<tr><td>chr8</td><td>66402389</td><td>66402426</td><td>19</td><td>AATGCCCTTTGATGCTAG</td><td>m||:|xxx||:x|:||x:</td><td>TCCTGCTGCTCCCTGTCC</td><td>M|||x||:|||x||:|x|</td><td>first-second</td><td>AATGCCCTTTGATGCTAGgtcaaaagggactttaaagGGACAGGGAGCAGCAGGA</td><td>−1.646440246</td></tr>
<tr><td>chr13</td><td>30941968</td><td>30942003</td><td>17</td><td>TCCCTCTCCCCACTGTTC</td><td>M||x|||x|x|x||:|||</td><td>TCCTTATAAAAACTGTAC</td><td>M||||x|xxxxx||:|x|</td><td>second-second</td><td>TCCCTCTCCCCACTGTTCcctttggtaaccacatcGTACAGTTTTTATAAGGA</td><td>−1.647319949</td></tr>
<tr><td>chr20</td><td>2514227</td><td>2514262</td><td>17</td><td>TCCCTCTGCCCACTGTGC</td><td>M||x|||:|x|x||:|x|</td><td>TCCTCCATCTCCCTCTAC</td><td>M|||x|xx|||x||x|x|</td><td>second-second</td><td>TCCCTCTGCCCACTGTGCtcagtcacttttccaaaGTAGAGGGAGATGGAGGA</td><td>−1.64776469</td></tr>
<tr><td>chr16</td><td>33585708</td><td>33585746</td><td>20</td><td>TCCTTCTACCCCCAGTTC</td><td>M||||||x|x|x|x:|||</td><td>CACTCCCCCACACTGTTC</td><td>mx||x|xx|x|x||:|||</td><td>second-second</td><td>TCCTTCTACCCCCAGTTCttcttttgctggatcttgtgGAACAGTGTGGGGGAGTG</td><td>−1.649265194</td></tr>
<tr><td>chr7</td><td>49800592</td><td>49800627</td><td>17</td><td>TATCCTGCTTTTTCCTCT</td><td>M||x||:|||x||x||xx</td><td>TCTTTCTGGTAACTGTTC</td><td>M|x||||:x|xx||:|||</td><td>first-second</td><td>TATCCTGCTTTTTCCTCTctttcatacaataccttGAACAGTTACCAGAAAGA</td><td>−1.658613522</td></tr>
<tr><td>chr9</td><td>53287100</td><td>53287139</td><td>21</td><td>TATTCTGCTTGTTCCTAC</td><td>M||x||:|||:||x||xx</td><td>TCTGCAGCTTACTGTTGA</td><td>Mx|:|x:|||xx|:x|:x</td><td>first-first</td><td>TATTCTGCTTGTTCCTACttatggttttacctggaacaaTCAACAGTAAGCTGCAGA</td><td>−1.659398296</td></tr>
<tr><td>chr15</td><td>48131996</td><td>48132028</td><td>14</td><td>AATGCTACTTGTCTCTGC</td><td>m||:||x|||:|xx||:x</td><td>TTCTCCTTCTCACTATTC</td><td>Mx||x||x|||x||x|||</td><td>first-second</td><td>AATGCTACTTGTCTCTGCtctctggctgtttaGAATAGTGAGAAGGAGAA</td><td>−1.659418994</td></tr>
<tr><td>chr21</td><td>47340484</td><td>47340515</td><td>13</td><td>AATGCTGCTTCATGCTGC</td><td>m||:||:|||xx|:||:x</td><td>GCCCCCTGCACCCTGTTC</td><td>m||xx||:|x|x||:|||</td><td>first-second</td><td>AATGCTGCTTCATGCTGCtctcttgggcacaGAACAGGGTGCAGGGGGC</td><td>−1.660990337</td></tr>
<tr><td>chr3</td><td>69069638</td><td>69069673</td><td>17</td><td>TAAGATGCTTGTTACTGC</td><td>M|x:x|:|||:||x||:x</td><td>TATGCATGTTATTACTGG</td><td>M||:|xxx||x||x||::</td><td>first-first</td><td>TAAGATGCTTGTTACTGCagataaggacatccaacCCAGTAATAACATGCATA</td><td>−1.661804303</td></tr>
<tr><td>chr6</td><td>83950371</td><td>83950402</td><td>13</td><td>TCATTCTGTTTGCTGCTC</td><td>M|x||||:x|x:||:x||</td><td>TCATTCTGCACACAGCTC</td><td>M|x||||:|x|x|x:x||</td><td>second-second</td><td>TCATTCTGTTTGCTGCTCccctaggaagaatGAGCTGTGTGCAGAATGA</td><td>−1.661984103</td></tr>
<tr><td>chr20</td><td>63491920</td><td>63491962</td><td>24</td><td>TCCTTCTGCACGCTGTAC</td><td>M||||||:|x|:||:|x|</td><td>GATAATGCTGATCCCTGG</td><td>m||xx|:||xx|xx||::</td><td>second-first</td><td>TCCTTCTGCACGCTGTACtctggccctggcccttcctctctaCCAGGGATCAGCATTATC</td><td>−1.662267288</td></tr>
<tr><td>chr29</td><td>36233940</td><td>36233981</td><td>23</td><td>CCCTGCCCCTCCCTCTCC</td><td>m|||x|xx|||x||x|x|</td><td>TTTCCTGCTTGTTGCTTG</td><td>Mx|x||:|||:||:||x:</td><td>second-first</td><td>CCCTGCCCCTCCCTCTCCtgcccactggcccttctctactcCAAGCAACAAGCAGGAAA</td><td>−1.669415976</td></tr>
</table>

chr21	47541355	47541395	22	CCCCTCTCCTCATTCTCC	m||x|||x|||xx|x|x|	TCATTCTACTAGATGTTC	M|x||||x||x:x|:|||	second-second	CCCCTCTCCTCATTCTCCcacaggacttcttgcccaataaGAACATCTAGTAGAATGA	−1.673713061
chr18	22709267	22709302	17	TCCTTCTTCTGGCTGGTC	M|||||x||x:||:x||	TCTTTCTTCTCCTTCTTC	M|x||||x|||xx|x|||	second-second	TCCTTCTTCTGGCTGGTCcatgctgtgacaagccaGAAGAAGGAGAAGAAAGA	−1.675416357
chr6	99234744	99234776	14	TATGCTACTTACAGCTGA	M||:||x|||xxx:||:x	TATGCTGGTTTCTGCTGT	M||:||:x||xx|:||:x	first-first	TATGCTACTTACAGCTGAtttcacactgttgtACAGCAGAAACCAGCATA	−1.679901484
chr16	54218594	54218626	14	ACCTTCTTCAACCAGTTC	m|||||x|xxx|x:|||	TCCTGCTGCTTGCTATTC	M|||x||:||x:||x|||	second-second	ACCTTCTTCAACCAGTTCacagaattccctgtGAATAGCAAGCAGCAGGA	−1.681498379
chr20	63491920	63491960	22	TCCTTCTGCACGCTGTAC	M|||||:|x|:||:|x|	TAATGCTGATCCCTGGTA	Mxx|x||:x||x||:x|x	second-second	TCCTTCTGCACGCTGTACtctggccctggcccttcctctcTACCAGGGATCAGCATTA	−1.682831461
chr14	18702881	18702912	13	CCCTTCTCCTCCCTGCTT	m|||||x|||x||:x|x	TACTCTGCTCCCTGCTGG	M|xx||:||xxx|:||::	second-first	CCCTTCTCCTCCCTGCTTgccccaccccaccCCAGCAGGGAGCAGAGTA	−1.684345721
chr10	53944535	53944565	12	TATAATTCTTGTTACTGT	M||xx|x|||:||x||:x	TCTGCTGACTGTTACAGG	Mx|:||:xx|:||x|x::	first-first	TATAATTCTTGTTACTGTgatgggacaagcCCTGTAACAGTCAGCAGA	−1.685275162
chr5	48841122	48841161	21	TCCTTCTACCCACTAGAA	M|||||x|x|x||xxxx	AATGCTGCTCATTACTGG	m||:||:||xx||x||::	second-first	TCCTTCTACCCACTAGAAacctctcttcctggggcttctCCAGTAATGAGCAGCATT	−1.685382489
chr19	61865750	61865791	23	TAAACTGCCTGTCGCTGG	M|xx||:|x|:|x:||::	CCCTTCTGCCCGCTTCTG	m|||||:|x|:||xx|x	first-second	TAAACTGCCTGTCGCTGGtttggtcctgcagggcccgccccCAGAAGCGGGCAGAAGGG	−1.68821855
chr19	61862023	61862064	23	TAAACTGCCTGTCGCTGG	M|xx||:|x|:|x:||::	CCCTTCTGCCCGCTTCTG	m|||||:|x|:||xx|x	first-second	TAAACTGCCTGTCGCTGGtttggtcctgcagggcccgccccCAGAAGCGGGCAGAAGGG	−1.68821855
chr1	58591023	58591057	16	TATTCTGCCTTCTGCTGG	M||x||:|x|xx|:||::	TCAACTGCTTTTTGCAGG	Mxxx||:|||x||:|x::	first-first	TATTCTGCCTTCTGCTGGttgtggaatcattttcCCTGCAAAAAGCAGTTGA	−1.691054421
chr13	75431798	75431834	18	TCCTTCTTCTCCCTCTTC	M|||||x|||x||x|||	GCTTTCTGCTTTCTGCCC	m|x||||:||xx||:xx|	second-second	TCCTTCTTCTCCCTCTTCccagggccccattgtgttGGGCAGAAAGCAGAAAGC	−1.697672972
chr16	44342005	44342037	14	TGCGCCGCTTGTAACTGG	Mxx:|x:|||:|xx||::	TCCCTTTTCTCGCTGTTA	M||x|x|x|||:||:||x	first-second	TGCGCCGCTTGTAACTGGgggagtactcctgtTAACAGCGAGAAAAGGGA	−1.699686409
chr29	37970505	37970546	23	TAATTCTCCTCTCTCTAC	Mxx||||x|||x||x|x|	CCCTTCTCCTCCCTGCCC	m|||||x|||x||:xx|	second-second	TAATTCTCCTCTCTCTACagaattggaactgtttgattctgGGGCAGGGAGGAGAAGGG	−1.700871563
chr28	33452964	33453004	22	TATCCTCTTTAGTTCTGG	M||x||xx||xx|x||::	GCCTTCTACCCGCTATTC	m|||||x|x|:||x|||	first-second	TATCCTCTTTAGTTCTGGgaggtggggctgggagtgagtgGAATAGCGGGTAGAAGGC	−1.702149177
chr27	41677119	41677157	20	TCTATTGTTTCTTGCTGC	Mx|xx|:x||x||:||:x	TACTTCTCATCACTATTC	Mx||||xx||x||x|||	first-second	TCTATTGTTTCTTGCTGCttgacccttgtggtttcagGAATAGTGATGAGAAGTA	−1.703103005
chr1	132784876	132784917	23	TCCCTCTGCTCTCCATTC	M||x|||:|||x|xx|||	TGAGCTGCCTCTTCCTGT	Mxx:||:|x|x||x||:x	second-first	TCCCTCTGCTCTCCATTCagtgccccctgctgggcagcatcACAGGAAGAGGCAGCTCA	−1.704053035
chr27	1880739	1880780	23	TCCTCCTGCTCTCTGTTT	M|||x||:|||x||:||x	TATTTTTACTTCCTGTTC	Mxx||x|x||xx||:|||	second-second	TCCTCCTGCTCTCTGTTTtcccctgtgttttattcccatcaGAACAGGAAGTAAAAATA	−1.705464772
chr4	93704	93745	23	TCCTTCTCCCAACAATTT	M|||||x|xxx|xx||x	TCCTTCTGCTCAATGCAC	M|||||:|||xx|:xx|	second-second	TCCTTCTCCCAACAATTTgtcttccaatgtatttgcctgttGTGCATTGAGCAGAAGGA	−1.705757774
chr1	113516176	113516211	17	TCTTTTTGCTCCCTCTTC	M|x||x|:|||x||x|||	TATGCTCTCAGTTGCTAA	M||:||xxxx:||:||xx	second-first	TCTTTTTGCTCCCTCTTCtttccctggcattgtttTTAGCAACTGAGAGCATA	−1.705910487
chr3	10038613	10038655	24	TCCCTTTGCTCTGTCTCC	M||x|x|:|||xx|x|x|	TATGCTCCTAGTTGCTAG	M||:||x||x:||:||x:	second-first	TCCCTTTGCTCTGTCTCCaactattttatatcaatcactatgCTAGCAACTAGGAGCATA	−1.706916884
chrX	39515474	39515508	16	ACCGCTGCTCTTTCCTGC	mxx:||:||xx||x||:x	CCCTTCTGCTCACTCTCC	m|||||:|||x||x|x|	first-second	ACCGCTGCTCTTTCCTGCttctcagaatctctctGGAGAGTGAGCAGAAGGG	−1.70706327
chr17	46699826	46699864	20	TCTTATGGTTGAAACTGG	Mx|xx|:x||:xxx||::	TCCTTCTGCTCTCTCTTC	M|||||:|||x||x|||	first-second	TCTTATGGTTGAAACTGGcttaaggagacaccctggcaGAAGAGAGAGCAGAAGGA	−1.710396428
chr9	79330776	79330808	14	GCCCTCTCCCCACTTTTC	m||x|||x|x|x||x|||	TGTGCTGCTTGTTCCAAG	Mx|:||:|||:||x|xx:	second-first	GCCCTCTCCCCACTTTTCtccaatgttctttcCTTGGAACAAGCAGCACA	−1.712715444
chr4	105171706	105171748	24	TATGCTTATTATTACTGT	M||:||xx||x||x||:x	TCCTCCTGCCCCCAATCC	M|||x||:|x|x|xx|x|	first-second	TATGCTTATTATTACTGTactaaaagactctgatgctgggagGGATTGGGGGCAGGAGGA	−1.712722896
chr2	21369570	21369608	20	GCCCTCTTCCCTCTGCAC	m||x|||x|x|x||:xx|	TCCTTCTGTTCCCTGTTC	M|||||:x||x||:|||	second-second	GCCCTCTTCCCTCTGCACgctgcctggcttccctcaggGAACAGGGAACAGAAGGA	−1.713088169
chr12	51510697	51510731	16	TCCACTGCCTGTCCCTGG	Mxxx||:|x|:|xx||::	CAAACTGCTTCCTGCTGG	m|xx||:|||xx|:||::	first-first	TCCACTGCCTGTCCCTGGcagtcacacatgcttgCCAGCAGGAAGCAGTTTG	−1.713550454
chr11	29386254	29386293	21	TCCTCCTCTTCCCCCTCC	M|||x||xx||x|xx|x|	TGTGCTGCTTGGTGCTGC	Mx|:||:|||:x|:||:x	second-first	TCCTCCTCTTCCCCCTCCccacagagtagggaggctttgGCAGCACCAAGCAGCACA	−1.714652601
chr13	24034579	24034620	23	TCCCTCTGCGAACTCTTC	M||x|||:|xxx||x|||	TGTGGTCCTTGTTACTAG	Mx|:x|x|||:||x||x:	second-first	TCCCTCTGCGAACTCTTCccttcctagtcactcaatcttttCTAGTAACAAGGACCACA	−1.715208362
chrX	39515467	39515508	23	TCTGCTGACCGCTGCTCT	Mx|:||:xxx:x|:||xx	CCCTTCTGCTCACTCTCC	m|||||:|||x||x|x|	first-second	TCTGCTGACCGCTGCTCTttcctgcttctcagaatctctctGGAGAGTGAGCAGAAGGG	−1.716734999
chr10	86600453	86600494	23	CCCTTCTTCTCTCTGATC	m|||||x|||x||:x||	TCCTGCTGCCTGCTATTT	M|||x||:|xx:||x||x	second-second	CCCTTCTTCTCTCTGATCttgttggagagctagtcctgggaAAATAGCAGGCAGCAGGA	−1.716851496
chr24	1369364	1369394	12	TTCTTCTGCCCACTGTCC	Mx|||||:|x|x||:|x|	CACCTCTGCTCTCTGGCC	mx|x|||:|||x||:xx|	second-second	TTCTTCTGCCCACTGTCCagctggccgcctGGCCAGAGAGCAGAGGTG	−1.717248751
chr7	38674758	38674788	12	TCATCTTCCTCGCTGTCC	M|x|xx|x|||:||:|x|	TCCTCCTGCCCGCTGGTG	M|||x||:|x|:||:x|x	second-second	TCATCTTCCTCGCTGTCCtccgcatgaactCACCAGCGGGCAGGAGGA	−1.717443279
chr11	62880572	62880611	21	TCTCTCTTCTCCCTTTTC	M|xx|||x|||x||x|||	TCTGCTGCTTCCTCCAGG	Mx|:||:|||xx|x|x::	second-first	TCTCTCTTCTCCCTTTTCcatcgctggagtaatccttagCCTGGAGGAAGCAGCAGA	−1.717758811
chrX	39515466	39515508	24	ATCTGCTGACCGCTGCTC	mx||x||:xx|:||:x||	CCCTTCTGCTCACTCTCC	m|||||:|||x||x|x|	second-second	ATCTGCTGACCGCTGCTCttcctgcttctcagaatctctctGGAGAGTGAGCAGAAGGG	−1.718355839
chr19	53051275	53051310	17	TCCTGCTCCTCCCTGTTC	M|||x||x|||x||:|||	ACTGGCCCTTGTGGCTGG	mx|:xxx|||:|x:||::	second-first	TCCTGCTCCTCCCTGTTCaggaccagcggctcaggCCAGCCACAAGGGCCAGT	−1.718597129
chr4	4824760	4824796	18	TATGGTTCTTATGAGTGG	M||:x|x|||x|xxx|::	TCCTCCTCCTCCCTGTTC	M|||x||x|||x||:|||	first-second	TATGGTTCTTATGAGTGGcatctgtgaactcccgagGAACAGGGAGGAGGAGGA	−1.719951862
chr16	72076319	72076352	15	TTTGCTGCTTGTTCATTT	Mx|:||:|||:||xx|xx	TCTACTGCTTGTCTCTGA	Mx|x||:|||:|xx||:x	first-first	TTTGCTGCTTGTTCATTTgttttgttgtctgccTCAGAGACAAGCAGTAGA	−1.722895459
chr14	78420668	78420704	18	CAAGCTGCGTGTTGCTGA	m|x:||:|x|:||:||:x	TCCCTCTGCTTCCTAACC	M||x|||:||xx||xxx|	first-second	CAAGCTGCGTGTTGCTGAggggagggtggagatgggGGTTAGGAAGCAGAGGGA	−1.724018525
chr4	80254326	80254367	23	TCTGCTGCTTATTTAGCA	Mx|:||:|||x||xxxxx	TCCGCTGCTTATTCCTGG	Mxx:||:|||x||x||::	first-first	TCTGCTGCTTATTTAGCAagctcttccagaatcagtcctttCCAGGAATAAGCAGCGGA	−1.724284511
chr11	77620797	77620837	22	TCCCTCTACTCACAGTTA	M||x|||x|||x|x:||x	TCCCTCTGATGGCCTTTC	M||x|||:x|x:|xx|||	second-second	TCCCTCTACTCACAGTTActgagtggtgctggggtcatgaGAAAGGCCATCAGAGGGA	−1.724570265
chr21	34193882	34193917	17	TAACCTGTTTCTTCCTGA	M|xx||:x||x||x||:x	TCCATCTCCTCACTTCTC	M||x|||x|||x||xx||	first-second	TAACCTGTTTCTTCCTGAcaggctggagggctggaGAGAAGTGAGGAGATGGA	−1.725643903

<table>
<tr><td>chr12</td><td>7376423</td><td>7376465</td><td>24</td><td>TATGCTACCTCTTGCTGA</td><td>M||:||x|x|x||:||:x</td><td>TAAGCACCCCGTCCCTGG</td><td>M|x:|xx|xx:|xx||::</td><td>first-first</td><td>TATGCTACCTCTTGCTGAgttctactgccaaattcccaggaaCCAGGGACGGGGTGCTTA</td><td>−1.7280273</td></tr>
<tr><td>chr4</td><td>71119963</td><td>71120002</td><td>21</td><td>CATGCTGCCTGGAACTGA</td><td>m||:||:|x|:xxx||:x</td><td>TTTGCTTCTTGCTGCTGA</td><td>Mx|:||x|||:x|:||:x</td><td>first-first</td><td>CATGCTGCCTGGAACTGAtgaggtcaggagaacccagttTCAGCAGCAAGAAGCAAA</td><td>−1.729542974</td></tr>
<tr><td>chr6</td><td>83647727</td><td>83647765</td><td>20</td><td>TCCATCTACTCACTGTCC</td><td>M||x|||x|||x||:|x|</td><td>TTTGCTAATTACAGATGG</td><td>Mx|:||xx||xxx:x|::</td><td>second-first</td><td>TCCATCTACTCACTGTCCatgaggactgttgtctgatcCCATCTGTAATTAGCAAA</td><td>−1.730747432</td></tr>
<tr><td>chr9</td><td>48821183</td><td>48821221</td><td>20</td><td>TCCTTCAGCTCACAATCC</td><td>M||||x:|||x|xx|x|</td><td>TCCTAATCCTCTCTCTTA</td><td>M|||xx|x|||x||x||x</td><td>second-second</td><td>TCCTTCAGCTCACAATCCtcctaaaaggtaactcaggcTAAGAGAGAGGATTAGGA</td><td>−1.731257184</td></tr>
<tr><td>chr7</td><td>6217331</td><td>6217362</td><td>13</td><td>CCCTTCTCCCCACTGTTC</td><td>m|||||x|x|x||:|||</td><td>TCCTTAGGCACCTGGTTC</td><td>M||||xx:|x|xxx:|||</td><td>second-second</td><td>CCCTTCTCCCCACTGTTCcatccacgcgtggGAACCAGGTGCCTAAGGA</td><td>−1.731772061</td></tr>
<tr><td>chr27</td><td>34802728</td><td>34802758</td><td>12</td><td>TCCATCTCCTCTTTGTTC</td><td>M||x|||x|||xx|:|||</td><td>CCACCCTGCTAGCTGTCC</td><td>m|xxx||:||x:||:|x|</td><td>second-second</td><td>TCCATCTCCTCTTTGTTCttctttcagaagGGACAGCTAGCAGGGTGG</td><td>−1.73194965</td></tr>
<tr><td>chr13</td><td>63949007</td><td>63949049</td><td>24</td><td>TCCATCTGCTCTCTATCC</td><td>M||x|||:|||x||x|x|</td><td>TAAGCTACCTGGCACTAG</td><td>M|x:||x|x|:xxx||x:</td><td>second-first</td><td>TCCATCTGCTCTCTATCCccagggatgccttgtaaagaaggcCTAGTGCCAGGTAGCTTA</td><td>−1.732794472</td></tr>
<tr><td>chr2</td><td>115621718</td><td>115621754</td><td>18</td><td>TCCTTCTCCTCACTGCTA</td><td>M|||||x|||x||:x|x</td><td>TATGCTACCCATTACACT</td><td>M||:||x|xxx||x|xxx</td><td>second-first</td><td>TCCTTCTCCTCACTGCTAttttgttttctcatttgaAGTGTAATGGGTAGCATA</td><td>−1.733551643</td></tr>
<tr><td>chr20</td><td>63491920</td><td>63491959</td><td>21</td><td>TCCTTCTGCACGCTGTAC</td><td>M|||||:|x|:||:|x|</td><td>AATGCTGATCCCTGGTAG</td><td>m||:||:x|xxx|:x|x:</td><td>second-first</td><td>TCCTTCTGCACGCTGTACtctggccctggcccttcctctCTACCAGGGATCAGCATT</td><td>−1.734287526</td></tr>
<tr><td>chrX</td><td>112274132</td><td>112274169</td><td>19</td><td>TCCTCCTCCTAGCTGCTC</td><td>M|||x||x||x:||:x||</td><td>TCCCTCTACTCTTCCTCC</td><td>M||x|||x|||xxxx|x|</td><td>second-second</td><td>TCCTCCTCCTAGCTGCTCtccatgatagtatgagagaGGAGGAAGAGTAGAGGGA</td><td>−1.734573366</td></tr>
<tr><td>chr2</td><td>122656558</td><td>122656595</td><td>19</td><td>CCGGCTGCTTGTACCTGA</td><td>mxx:||:|||:|xx||:x</td><td>CCCTCCTACTCCCTGCTC</td><td>m|||x||x|||x||:x||</td><td>first-second</td><td>CCGGCTGCTTGTACCTGAgcaagtctcttcttcccgaGAGCAGGGAGTAGGAGGG</td><td>−1.736016154</td></tr>
<tr><td>chr24</td><td>9585619</td><td>9585658</td><td>21</td><td>TATGCTTCTAGTTCCTGT</td><td>M||:||x||x:||x||:x</td><td>TAGGATGCTTGCTCCTTG</td><td>M|x:x|:|||:x|x||x:</td><td>first-first</td><td>TATGCTTCTAGTTCCTGTaaaatgtcaaactgaaggtgaCAAGGAGCAAGCATCCTA</td><td>−1.737121474</td></tr>
<tr><td>chr7</td><td>3844259</td><td>3844300</td><td>23</td><td>CAAGCTCCATGGTGCTAC</td><td>m|x:||x|x|:x|:||xx</td><td>TCCTTCTCCTCTCTCTCC</td><td>M|||||x|||x||x|x|</td><td>first-second</td><td>CAAGCTCCATGGTGCTACcaaaaaaattttaaaaaagagagGGAGAGAGAGGAGAAGGA</td><td>−1.740447436</td></tr>
<tr><td>chr1</td><td>118200016</td><td>118200046</td><td>12</td><td>TATCCTGCTTGCTACATG</td><td>M||x||:|||:x|x|xx:</td><td>TCCCCTCATTGTTCCTGG</td><td>Mxxx||xx||:||x||::</td><td>first-first</td><td>TATCCTGCTTGCTACATGggagcagggttgCCAGGAACAATGAGGGGA</td><td>−1.742424533</td></tr>
<tr><td>chr16</td><td>51979301</td><td>51979341</td><td>22</td><td>TCCTCCTCCTCTCTGTTC</td><td>M|||x||x|||x||:|||</td><td>TAACCATCTTGTTGGAGG</td><td>M|xx|xx|||:||:xx::</td><td>second-first</td><td>TCCTCCTCCTCTCTGTTCtataaaagactctggcacccagCCTCCAACAAGATGGTTA</td><td>−1.747280437</td></tr>
<tr><td>chrX</td><td>3046636</td><td>3046678</td><td>24</td><td>TCCTTCTGCTCTCTGTGC</td><td>M|||||:|||x||:|x|</td><td>GTTGCTCCCTATTTCGGG</td><td>mx|:||x|x|x||x|x::</td><td>second-first</td><td>TCCTTCTGCTCTCTGTGCcctgagagtgataagtaactacttCCCGAAATAGGGAGCAAC</td><td>−1.747744765</td></tr>
</table>

First RVD sequence: NI-NG-NN-HD-NG-NN-HD-NG-NG-NN-NG-NG-NN-HD-NG-NN-NN

Second RVD sequence: HD-HD-NG-NG-HD-NG-NN-HD-NG-HD-NN-HD-NG-NN-NG-NG-HD

N-Terminal first: false

N-Terminal second: false

Hetero-dimers only: false

Architecture: min=12, max=24

Filter: q=0.4

RVD specificities: NA

Maximum number of targets: 100

Output: GF

Table S2 Summary of SNPs and indels.

chrom	stat_loci	end_loci	space_length	score	SNP	SNP_effects	INDEL	INDEL_effects
chr1	58591023	58591057	16	-1.69105442	0		0	
chr1	1.14E+08	1.14E+08	17	-1.70591049	1	intergenic	0	
chr1	1.18E+08	1.18E+08	12	-1.74242453	0		0	
chr1	1.33E+08	1.33E+08	23	-1.70405303	0		0	
chr1	1.33E+08	1.33E+08	16	-1.59797137	0		0	
chr1	1.52E+08	1.52E+08	21	-1.64380622	0		0	
chr10	53944535	53944565	12	-1.68527516	0		0	
chr10	86600453	86600494	23	-1.7168515	1	intron	0	
chr11	29386254	29386293	21	-1.7146526	0		0	
chr11	62880572	62880611	21	-1.71775881	1	intergenic	0	
chr11	77620797	77620837	22	-1.72457027	1	intergenic	0	
chr11	86404351	86404388	19	-1.63097977	0		0	
chr12	7376423	7376465	24	-1.7280273	0		1	intergenic
chr12	51510697	51510731	16	-1.71355045	0		0	
chr12	68932365	68932404	21	-1.58833279	2	intergenic	0	
chr13	17132488	17132528	22	-1.60301088	1	intergenic	0	
chr13	24034579	24034620	23	-1.71520836	0		0	
chr13	30941968	30942003	17	-1.64731995	0		0	
chr13	63949007	63949049	24	-1.73279447	1	intron	0	
chr13	75431798	75431834	18	-1.69767297	0		0	
chr14	18702881	18702912	13	-1.68434572	0		0	
chr14	37026557	37026591	16	-1.60478917	1	intron	0	
chr14	78168541	78168581	22	-1.53674942	0		0	
chr14	78420668	78420704	18	-1.72401852	2	intergenic	0	
chr15	23913129	23913170	23	-1.59702431	1	intergenic	0	
chr15	48131996	48132028	14	-1.65941899	0		0	
chr15	69632598	69632632	16	-1.54213412	0		0	
chr16	33585708	33585746	20	-1.64926519	0		0	
chr16	44342005	44342037	14	-1.69968641	0		0	
chr16	51979301	51979341	22	-1.74728044	0		0	
chr16	54218594	54218626	14	-1.68149838	1	intergenic	0	
chr16	72076319	72076352	15	-1.72289546	0		0	
chr17	12117522	12117554	14	-1.44814736	0		0	
chr17	32490313	32490347	16	-1.43066557	1	intergenic	0	
chr17	46699826	46699864	20	-1.71039643	0		0	
chr17	51217650	51217686	18	-1.64071905	0		0	

chr17	53679578	53679619	23	-1.60699223	1	intron	0	
chr18	22709267	22709302	17	-1.67541636	2	intergenic	0	
chr19	53051275	53051310	17	-1.71859713	1	intergenic	0	
chr19	61862023	61862064	23	-1.68821855	0		0	
chr19	61865750	61865791	23	-1.68821855	0		0	
chr2	6313955	6313991	18	-0.49385249	0		0	
chr2	21369577	21369608	13	-1.40357807	0		0	
chr2	1.16E+08	1.16E+08	18	-1.73355164	1	intron	0	
chr2	1.23E+08	1.23E+08	19	-1.73601615	3	intergenic	0	
chr20	2514227	2514262	17	-1.64776469	0		0	
chr20	8292741	8292775	16	-1.47997815	1	intergenic	0	
chr20	63491920	63491962	24	-1.66226729	3	intron	1	intron
chr21	12050595	12050631	18	-1.57462717	0		1	intergenic
chr21	34193882	34193917	17	-1.7256439	0		0	
chr21	47340484	47340515	13	-1.66099034	0		0	
chr21	47541355	47541395	22	-1.67371306	0		0	
chr24	1369364	1369394	12	-1.71724875	0		0	
chr24	9585619	9585658	21	-1.73712147	0		0	
chr26	28667329	28667364	17	-1.63536425	2	intergenic	0	
chr26	28740108	28740150	24	-1.58601299	0		0	
chr27	1880739	1880780	23	-1.70546477	1	intergenic	0	
chr27	34802728	34802758	12	-1.73194965	0		0	
chr27	41677119	41677157	20	-1.70310301	1	intergenic	0	
chr28	33452964	33453004	22	-1.70214918	0		0	
chr29	36233940	36233981	23	-1.66941598	0		0	
chr29	37970505	37970546	23	-1.70087156	0		0	
chr3	10038613	10038655	24	-1.70691688	1	intergenic	1	intergenic
chr3	22313921	22313952	13	-1.55419318	0		0	
chr3	39762888	39762922	16	-1.61359466	0		0	
chr3	69069638	69069673	17	-1.6618043	2	intergenic	0	
chr4	93704	93745	23	-1.70575777	1	intergenic	1	intergenic
chr4	4824760	4824796	18	-1.71995186	1	intergenic	0	
chr4	71119963	71120002	21	-1.72954297	0		0	
chr4	80254326	80254367	23	-1.72428451	1	intergenic	0	
chr4	1.05E+08	1.05E+08	24	-1.7127229	0		0	
chr5	48841122	48841161	21	-1.68538249	0		0	
chr6	35405629	35405665	18	-1.60832042	1	intron	1	intron
chr6	60529316	60529353	19	-1.62458732	1	intergenic	0	
chr6	83647727	83647765	20	-1.73074743	1	intergenic	0	
chr6	83950371	83950402	13	-1.6619841	1	intron	0	

chr6	99234744	99234776	14	-1.67990148	0		0
chr7	3844259	3844300	23	-1.74044744	1	intergenic	0
chr7	6217331	6217362	13	-1.73177206	1	intron	0
chr7	49800592	49800627	17	-1.65861352	0		0
chr7	99559623	99559657	16	-1.63326407	0		0
chr8	66402389	66402426	19	-1.64644025	1	intergenic	0
chr9	15855225	15855255	12	-1.59217359	1	intergenic	0
chr9	48821183	48821221	20	-1.73125718	1	intergenic	0
chr9	53287100	53287139	21	-1.6593983	1	intron	0
chr9	79330776	79330808	14	-1.71271544	0		0
chrX	3046636	3046678	24	-1.74774476	0		0
chrX	39515467	39515508	23	-1.716735	0		0
chrX	39515474	39515508	16	-1.70706327	0		0
chrX	1.12E+08	1.12E+08	19	-1.73457337	0		0

Table S3 The target sites of TALENs.

Target site	Leftarm and rightarm
MSTN-T1	Leftarm:ATGCTGCTTGTTGCTGG<br>RVD:NI NG NN HD NG NN HD NG NG NN NG NG NN HD NG NN NN<br>Rightarm:CCTTCTGCTCGCTGTTC<br>RVD:HD HD NG NG HD NG NN HD NG HD NN HD NG NN NG NG HD
MSTN-T2	Leftarm:TTGCCCAAGGCTCCTCC<br>RVD:NG NG NN HD HD HD NI NI NN NN HD NG HD HD NG HD HD<br>Rightarm:GGACATCGTACTGATCA<br>RVD:NN NN NI HD NI NG HD NN NG NI HD NG NN NI NG HD NI

Table S4 The primers for q-PCR.

Gene		primers sequence (5’ to 3’)	TM (ºC)	Efficiency
MSTN	F	GTGTTGCAAAACTGGCTCAA	59	94.20%
	R	TCATCACAATCAAGCCCAAA		
ACVR2B	F	AGAGTGACCTCACTGCTG	59	95.60%
	R	TCATCCACAGGTCCGTCG		
FST	F	GGGACTTCAAGGTTGGCAG	59	101.90%
	R	TTCACCTTCCTCCTCGTCC		
MYF6	F	CTTGAGGGTGCGGATTTCC	59	96.80%
	R	TCTCCACTACCTCCTCCACG		
P21	F	CAGAAGAGCCACAGGTGC	59	104.10%
	R	CGTCTCGGTGACAAAGTCG		
SMAD2	F	ACCAGGTCTCTAGATGGTCG	59	98.30%
	R	GGGCAGAACTGGTGTCTC		
SMAD3	F	GCAAGATTCCACCAGGGTGC	59	96.00%
	R	CAGGTGCAGCTCAATCCAG		
MYOD	F	GTGCAAACGCAAGACGACTAAC	59	98.60%
	R	TGGTTTGGGTTGCTAGACGTG		
MYOG	F	CAGTGAATGCAGCTCCCATAGTG	59	96.3 %
	R	AGGTGAGGGAGTGCAGATTGTG		
GAPDH	F	ATGTTTGTGATGGGCGTGA	59	93.80%
	R	AAGCAGGGATGAAGTTCTGG		

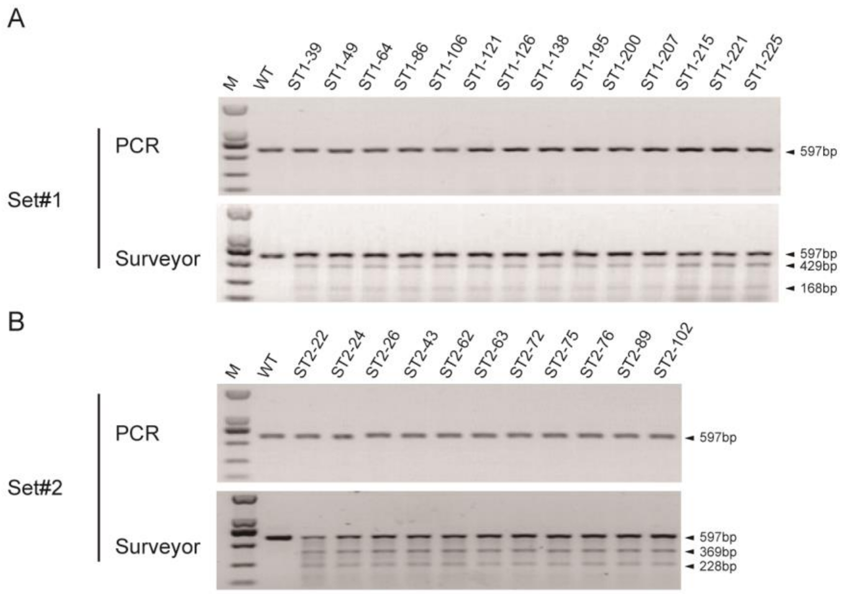

Figure S1. Identification of the cell clonies harbored the *MSTN* gene mutation in *MSTN*-T1 (A) and *MSTN*-T2 (B) by PCR and T7EI assay. The detection of the *MSTN* gene in cell clonies by PCR. The genomic regions surrounding the target site were amplified and a 597 base pair PCR product of the *MSTN* gene was obtianed. Genotyping of *MSTN* mutant lamb by the T7EI assay. *MSTN* gene of each cell clonies is assayed and presented in the same order as PCR. Individuals with one band of wild-type (WT) and mutated alleles have three bands in T7EI assay.

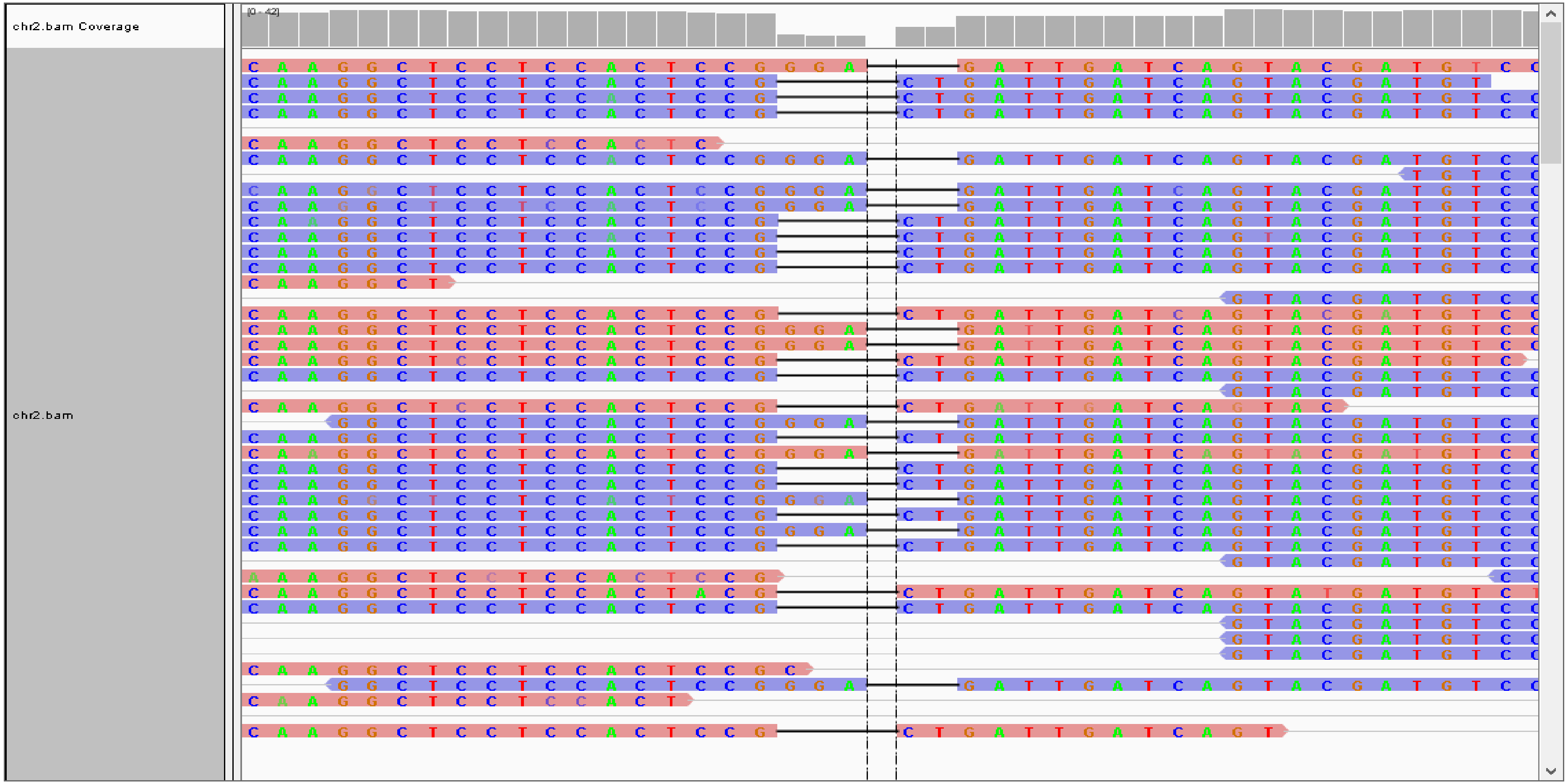

Figure S2. The results of sequence.

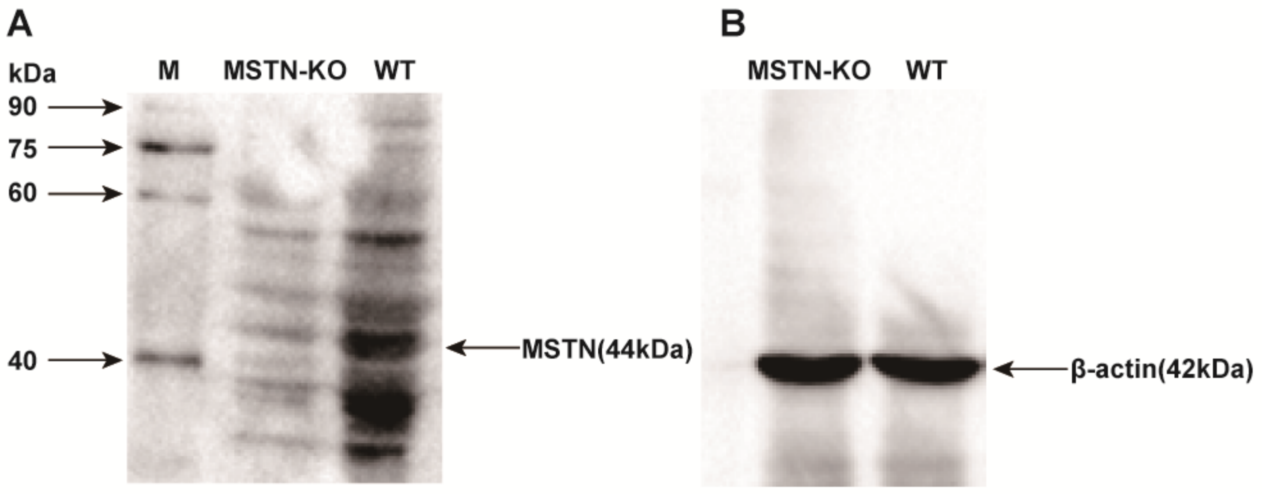

Figure S3. The full-length blots of Figure 2E
